# D-Mannoside FimH Inhibitors as Non-Antibiotic Alternatives for Uropathogenic *Escherichia coli*

**DOI:** 10.3390/antibiotics10091072

**Published:** 2021-09-04

**Authors:** Alfredo Montes-Robledo, Rosa Baldiris-Avila, Johan Fabian Galindo

**Affiliations:** 1 Grupo de Investigación Microbiología Clínica y Ambiental, Facultad de Ciencias Exactas y Naturales, Universidad de Cartagena, Cartagena de Indias 13001, Colombia; amontesr@unicartagena.edu.co; 2 Maestría en Microbiología, Facultad de Medicina, Universidad de Cartagena, Cartagena de Indias 13001, Colombia; 3 Grupo de Investigación CIPTEC, Facultad de Ingeniería, Fundacion Universitaria Tecnologico Comfenalco—Cartagena, Cartagena de Indias 13001, Colombia; 4 Departamento de Química, Universidad Nacional de Colombia, Bogotá 11321, Colombia

**Keywords:** UPEC, FimH, molecular dynamics, virulence factors, antibiotic resistance

## Abstract

FimH is a type I fimbria of uropathogenic *Escherichia coli* (UPEC), recognized for its ability to adhere and infect epithelial urinary tissue. Due to its role in the virulence of UPEC, several therapeutic strategies have focused on the study of FimH, including vaccines, mannosides, and molecules that inhibit their assembly. This work has focused on the ability of a set of monosubstituted and disubstituted phenyl mannosides to inhibit FimH. To determine the 3D structure of FimH for our in silico studies, we obtained fifteen sequences by PCR amplification of the *fimH* gene from 102 UPEC isolates. The *fimH* sequences in BLAST had a high homology (97–100%) to our UPEC *fimH* sequences. A search for the three-dimensional crystallographic structure of FimH proteins in the PDB server showed that proteins 4X5P and 4XO9 were found in 10 of the 15 isolates, presenting a 67% influx among our UPEC isolates. We focused on these two proteins to study the stability, free energy, and the interactions with different mannoside ligands. We found that the interactions with the residues of aspartic acid (ASP 54) and glutamine (GLN 133) were significant to the binding stability. The ligands assessed demonstrated high binding affinity and stability with the lectin domain of FimH proteins during the molecular dynamic simulations, based on MM-PBSA analysis. Therefore, our results suggest the potential utility of phenyl mannoside derivatives as FimH inhibitors to mitigate urinary tract infections produced by UPEC; thus, decreasing colonization, disease burden, and the costs of medical care.

## 1. Introduction

Urinary tract infections (UTIs) are one of the main causes of visits to health centers around the world, with uropathogenic *Escherichia coli* (UPEC) being the main bacterial agent associated with UTIs [1]. UTIs can be transmitted through the community (~90%) as well as hospitals (~50%), and are associated with high rates of morbidity [2]. UPEC strains can colonize urinary tissue and cause pathogenesis through diverse virulence factors, which are present in the chromosome or in mobile extrachromosomal material, such as plasmids [3]. Virulence factors play an important role in bacterial isolates because they allow bacteria to colonize the urinary tract and persist despite the host’s defense mechanisms [4].

UPEC strains can be classified as pathogenic based on predictive molecular markers, such as phylogenetic assignation, virulence factors, and antibiotic resistance of the isolates [5]. These markers play a role in the adhesion, invasion, and formation of intracellular bacterial communities in the uroepithelium, which results in a high frequency of recombination, acquisition, and/or loss of genetic information through horizontal genetic transfer. They are also useful as descriptors of the great genetic diversity and genome plasticity, accelerating the adaptation of UPECs to new ecological niches [6,7]. UTIs present high morbidity due to bacterial antibiotic resistance, which has increased over time due to antibiotic overuse. Therefore, it is necessary to look for new non-antibiotic alternatives to mitigate these infections. The pathogenicity mechanism of UPEC begins with cellular adhesion through the participation of fimbrial adhesins, and other virulence genes present can aid the process [8,9].

The *fimH* gene encodes a protein of approximately 300 amino acids (FimH), which participates in the regulation and mediation of fimbriae. It is composed of two domains: a pilin domain that allows polymerization [10] and a lectin domain, which allows the binding to the host cells through mannosylated proteins. These proteins are present in the bladder epithelium and bind to FimH based on the rearrangement of the host actin cytoskeleton [11]. Due to their role in the virulence of UPEC, several therapeutic strategies have focused on FimH, including vaccines, mannosides (as competitive compounds of the FimH binding pocket), and molecules which inhibit the assembly of FimH. Studies have shown that FimH inhibitors can also increase the susceptibility of UPEC to antimicrobials even in resistant bacteria, making them a prospective non-antibiotic strategy for UPEC management and treatment [12,13].

Currently, different contributions have been reported that show that several inhibitory molecules function as potential candidates with the ability to bind or couple to FimH in different states of low, medium, and high affinity [14,15,16]. These contributions have also focused on the development of bioavailable mannosides molecules with an anti-virulence behavior, suggesting that the compounds derived from D-mannose show a high efficacy both in reducing symptoms and in the rate of recurrence of urinary infections [17,18]. In addition, Sarks et al. [19] suggest a bioavailable vaccine, which can induce functional antibodies over patients with recurrent urinary tract infections, contributing to the prevention of urinary infections. However, more accurate, reproducible, and standardized tests are needed to explore the effectiveness of D-mannose-derived antagonists in FimH.

In Colombia, there are few studies showing the characteristics of UPEC strains and the possible FimH proteins where these bacterial strains are present; therefore, the objectives of the present work are: (i) to identify and characterize strains of UPEC through phylogenetic analysis, different virulence factors, and antibiotic resistance genes, (ii) to sequence the *fimH* gene and to select the protein structures from the Protein Data Bank (PDB) for further in silico studies, (iii) to perform Molecular Docking and Molecular Dynamics (MD) simulations with a set of inhibitory molecules of fimbriae type 1 (FimH), and (iv) to complete an atomic characterization of the receptor–ligand type interactions, identifying possible inhibitor candidates with the lowest free energy interaction. This characterization can be helpful to advance in the design of FimH inhibitory molecules to treat urinary tract infections produced by UPEC.

## 2. Results and Discussion

### 2.1. Bacterial Strains, Genotypic Confirmation, and Phylogenetic Analysis

One hundred and two different UPEC strains were confirmed based on their morphological, genotypic, and physiological properties. The UPEC strains were assigned into phylogroups A (14.7%), B1 (5.0%), B2 (23.5%), C (9.8%), D (26.4%), E (1.0%), F (3.9%), Clade I (7.8%), and unknown (6.8%). The phylogenetic distribution of the strains may be related to their geographical distribution, intrinsic virulence, bacterial traits, and ecological source [20,21]. In this work, we observed a high prevalence of groups D and B2 which can be attributed to the strains associated to diseases outside the gastrointestinal tract. The presence of these different phylogroups can be related to the migration of the strains towards the urethra or other organs, causing different infections such as urinary tract infections, neonatal meningitis, sepsis, pneumonia, and surgical site infections, as well as infections in other extraintestinal sites [22]. These findings are consistent with UPEC strains of phylogroups B2 and D, presenting more virulence genes among patients with urinary infection. For this reason, it is necessary to take measures to combat these virulent strains through the design and implementation of prevention strategies and the search for possible drug candidates to better manage UTIs [23].

### 2.2. Detection of Virulence Factors and Biofilm-Forming Ability

The prevalence of virulence factors and biofilm-forming capacity are shown in Table 1, where the most prevalent virulence genes were *fimH* (95%), *fyuA* (84%), *chuA* (69%), and *kpsMTII* (68%). These genes were present in more than 50% of the strains analyzed; therefore, they can be considered molecular predictors of UPEC [24,25]. Furthermore, these virulence factors could be potential targets for the development of new non-antibiotic antibacterial therapies, including the development of potential vaccines which could help to prevent the colonization of the urinary tract by UPEC strains. According to our results, all UPEC strains presented at least one virulence factor, of the eight tested, and 92% of them have two or more. UPEC strains presenting several virulence factors can behave as intracellular pathogens, taking advantage of the host’s behavior and susceptibility [25]. Taking a closer look at the prevalence of the virulence factors studied across the different phylogenetic groups, we found a strong correlation (*p*-value 0.0002) with the *fimH*, *chuA*, *kpsMTII*, and *YjaA* genes. This relationship between the molecular markers and phylogroups supports that bacterial traits (phylogenetic group and virulence factors) are a result of the different origins of the samples and their ecological source. Thus, the virulence factors can work as molecular predictors of UPEC strains [8,24,25,26].

#### Biofilm-Forming Abilities and Congo Red Agar Method

Biofilm formation was detected in 95% (*p*-value 0.0195) of the strains evaluated, displaying production and formation of fully established biofilms based on the microtiter assay, which was stained with violet crystal. This three-dimensional extracellular matrix could limit the access of antibacterial agents, antibodies, and white blood cells. Additionally, the proximity of cells within a biofilm can facilitate the exchange of genetic material (virulence genes, resistance genes, plasmids, transposons, and integrons) and accelerate the spread of antimicrobial resistance [27]. Biofilm formation was also closely related to the different phylogroups (*p*-value 0.0001). This characteristic provides plasticity to the bacterial strains and may influence the virulence and the bacterial fitness to persist in hostile environments. The Congo Red tests, which evaluate the sugar production capability of the biofilm (aggregative fimbriae and/or cellulose), were positive for all isolates (*p*-value 0.001). We also found that the high production of aggregate fimbriae is due to the strong and/or moderate formation of biofilms; thus, functional microbial amyloids contribute to the natural ability to coordinate the assembly of the cellular extra-polysaccharide matrix, as well as to the adhesion and final biofilm formation in hostile environments with low nutrient capacity [28,29].

### 2.3. Antimicrobial Susceptibility of Isolated UPEC

After assessing the pattern of susceptibility of the isolates to 20 antibiotics, the highest resistance indices were observed for beta-lactams, quinolones, and cephalosporins (Table 2). Machado-Alba et al. [30] demonstrated that the use and prescription of antibiotics is provided according to the geographical area in Colombia, and it is common to prescribe only one antibiotic in the capital cities, while it is more common to prescribe two or more antibiotics in the municipalities. The UPEC strains of this study showed a high resistance rate to ampicillin, to cefepime, to cirprofloxacin, and to cefoxitin, and this high rate of bacterial resistance can be attributed to the use of first-generation cephalosporins as the initial treatment schemes at the hospitals in Colombia. In this study, a high rate of resistance to ciprofloxacin was also observed, and it can be attributed to the use of the antibiotic in the non-prescription community or by mutations in the quinolone determination resistance (QRDR) regions of the *gyrA* and *parC* genes [31,32].

Additionally, 54% of the evaluated *E. coli* strains showed a MAR index ≥ 0.2, which indicates a multi-resistant strain. Another cause of bacterial resistance is the production of ESBL enzymes, observed in 26% of the isolates. In addition, bacterial strains can acquire or transmit gene resistance over time, complicating the management and control of MDR and ESBL strains. In this study, we observed that the UPEC strains expressed the TEM (50%), *OXA* (19.3%), *SHV* (16.7%), and *CTX-M-1* (3.9%) genes, which could explain why the emergence of *TEM*, *SHV,* and *CTX-M* enzymes has become a serious clinical problem worldwide, and in particular during the last decade [33]. The presence of this high rate of resistance in UPEC strains producing ESBL can be attributed to the uncontrolled use and consumption of antibiotics in the community, resulting in a greater capacity for dissemination and persistence due to the acquisition and transfer of β-lactamase genes [34]. This is the main reason why studies are underway in order to find a new non-antibiotic strategy, including therapies or treatments targeting polysaccharide capsules, flagella, pili, curli, adhesins, outer-membrane proteins, as well as secreted toxins, secretion systems, and iron-uptake receptors [8]. In this work, we propose a new non-antibiotic alternative against UPEC, which could be able to counteract the bacterial burden associated with the disease.

### 2.4. In Silico Evaluation of the Previously Characterized UPEC Isolates

Based on the previous analysis of the UPEC strains, we selected fifteen isolates for further study according to characteristics such as phylogenetic distribution, multiple antibiotic resistance, and presence or absence of virulence factors (Table 3). The two selected FimH proteins (4X09 and 4X5P PDB codes) are not associated with a particular UPEC strain, phylogroup, virulence factor, or antibiotic susceptibility; however, they are present in all the isolates. It is known that to date, there are more than one hundred fimH crystallographic structures reported; however, in this study, these two proteins mentioned above were the prevalent ones in the clinical strains of this contribution.

#### Molecular Sequences of the *fimH* Gene of Uropathogenic Strains

The consensus sequences, without noise and formatting, were translated from nucleotides to amino acids, taking into account the percentage of identity for all isolates in a range of 97–100%. Hasanzadeh et al. [35] confirm that the *fimH* sequence in UPEC strains maintains a 97% similarity percentage based on the results of the sequence homology of the *fimH* gene; thus, this urovirulent gene is associated with genetic variants and, therefore, could be clinically relevant. Nevertheless, it is known that the *fimH* gene is subjected to a strong selective pressure and it is likely to show a high degree of sequence heterogeneity contributing to a more precise characterization of the UPEC strains [36]. Once the sequences were verified and confirmed, a search for the three-dimensional crystallographic structure of the FimH proteins on PDB gave us the following results: Protein 1: 4X5P, Protein 2: 4XO9, Protein 3: 4XOD, Protein 4: 4XOE, and Protein 5: 5JQI. The proteins with codes 4XO9 and 4X5P were the most representative, with a 67% prevalence in the sequenced isolates. Thus, we selected these two proteins for the in silico assays. These results suggest that UPEC strains present a conserved sequence for FimH proteins independent of the pheno-genotypic characteristics of the *E. coli* strains.

### 2.5. Molecular Docking

The root-mean-square deviation (RMSD) value between the protein and the native binder was 0.096 Å. A molecular coupling is considered adequate, and its conformation reliable, if the RMSD value is less than 2.0 Å [37]; therefore, the set of couplings in this study is reliable. A summary of the best conformations obtained from the molecular coupling study are shown in Table 4.

**Figure 1:**
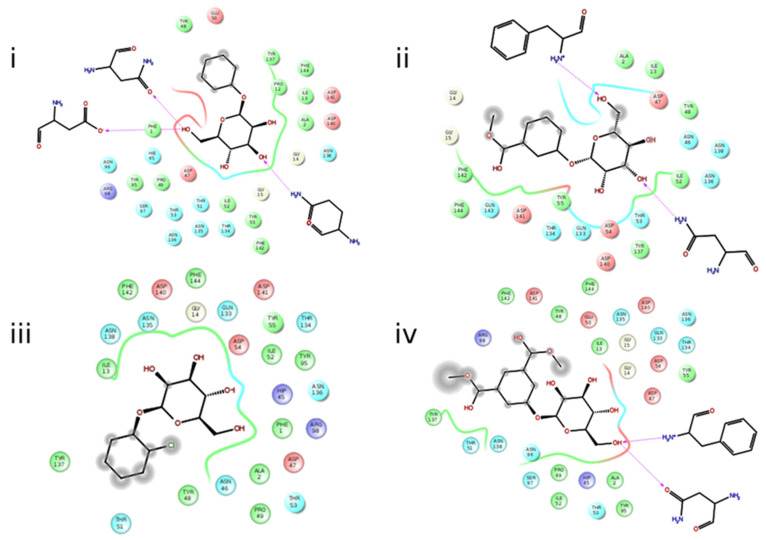
Interactions between proteins and ligands. (**i**) Interaction between protein 4XO9 and ligand **b**. (**ii**) Interaction between protein 4XO9 and ligand g. (**iii**) Interaction between 4X5P protein and ligand e. (**iv**) Interaction between 4X5P protein and ligand j.

To choose the best out of the nine poses of the binder within the protein (Supplementary Materials Tables S1–S20), two criteria were considered: the lowest energy and the lowest root-mean-square deviation (RMSD) with respect to the native binder. These couplings provide a static view of the different interactions within the complex (protein–ligand) that contribute to the binding affinity. For example, in protein 4XO9, the residues of aspartic acid (ASP 54), asparagine (ASN 46), and glutamine (GLN 133) form hydrogen bonds with the hydroxyl groups of the mannopyranose of ligand b (Figure 1). In the same protein, ligand g has an interaction with the phenylalanine (PHE 1) and aspartic acid (ASP 135) residues. In the case of protein 4X5P, ligand j interacted with phenylalanine (PHE 1) and asparagine (ASN 46) so that the binding affinities of D-mannose inhibitors are exceptionally good [38]. All these interactions are important for the stabilization of the protein–ligand complex, with hydrogen bonds usually constituting one of the most important contributions to the total interaction energy in the absence of covalent ligand–receptor bonds.

Alam et al. [39] evidenced that the hydrogen bonds were formed mostly with the waste Asp140, Gln133, Asn135, and Phe1, despite having different link distances, which coincides largely with the data found in this study. This result confirms that the hydrogen bonds play a vital role in the configuration of the specificity of binding between the ligand and the receptor. Therefore, these interactions become important in the drug design of chemical and biological processes, molecular recognition, and biological activity. Additionally, these residues play a promising role in some catalytic sites of FimH proteins responsible for various metabolic activities of UPEC strains. These interactions also act as strong anchor points to maintain the structural integrity of the ligand–protein complex [40]. The conformational changes determine the intrinsic activity of the complex. After the docking analysis, we performed a molecular dynamics simulation to better understand the stability of the hydrogen bond interactions and to estimate the binding energy of the ligand–protein complex.

### 2.6. Molecular Dynamics Simulations

The behavior of the complexes was studied using a 200 ns molecular dynamics simulation in aqueous solution. The stability throughout the simulation of the complexes formed with the different ligands and the native ligand of each of the proteins was monitored using the RMSD as a function of time. When analyzing the RMSD graphs of the systems, a relative equilibrium was observed during the 200 ns of simulation time (Supplementary Materials Figures S1–S22), indicating that the results obtained from the coupling model are reasonable and valid. The RMSD of the alpha carbon skeleton atoms (Cα) of the initial conformation of the protein–ligand complexes were plotted as a function of time. In particular, the simulations revealed good structural integrity for all the complexes with proteins 4X5P and 4XO9 respectively, attributable to the hydrogen bond interactions between aspartic acid 54 and the mannopyranose of the ligands. We also found high Van der Waals interactions, which can be due to a hydrophobic edge interaction of the FimH binding pocket, although the amino acids involved in the interaction were not the same for all the complexes. These interactions remained in relative equilibrium during the dynamics and as a consequence are more likely to also interact when tested in vivo. The stability of the receptor–ligand complex is measured by conformational changes in the dynamic behavior of the complex [41,42,43]. RMSD values can vary throughout the stabilization process of the complex, which contributes to identifying the interaction or binding coupling between FimH proteins and binders [44]. Consequently, the RMSD data from the complexes show the most reliable binding trajectories between the FimH proteins and the binders tested.

The free binding energies were calculated for all the native-protein (4XO9 and 4X5P) and ligand–protein (b to k) complexes using the MM-PBSA/MM-GBSA methods. Dumych et al. [45] reported that the Poisson–Boltzmann free binding energy (MM-PBSA) method shows the same trend as in vivo tests. In both cases, the native-protein complexes have a negative free energy, which is consistent with the fact that experimentally, it is possible to observe these complexes. In order to develop a new inhibitor, it is necessary to have a lower free energy for the ligand–protein complex in comparison to the native-protein complex. Based on our simulations, the strongest binders to 4XO9 were ligands b, f, and g (Table 5), while the strongest binders to 4X5P were ligands **e**, **i**, and **j** (Table 6). These results suggest that D-mannose-derived ligands are good candidates to target inhibition of the FimH proteins in UPEC. Regardless of the FimH variants, the inhibitors derived from D-mannose have a high affinity because the structures of the FimH receptor binding domain in complex with mannose strongly interact with all its hydroxyl groups [46].

**Figure 2:**
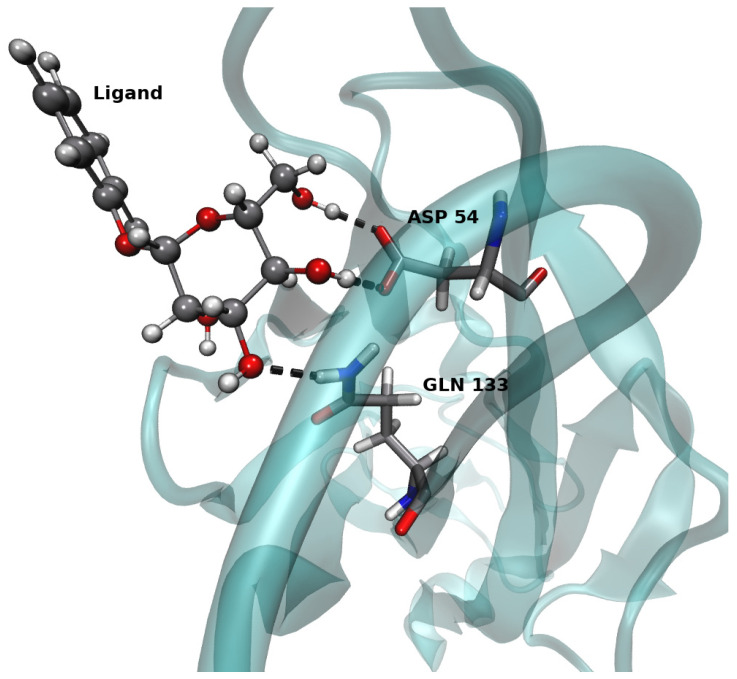
Visualization of the hydrogen bonds between ASP 54, GLN 133 residues, and ligand e.

In addition to the contribution to the stability of the complex made by the ASP 54 hydrogen bond interaction, we also observed, for the strongest binders, an interaction between the ligand and GLN 133 (Table 7). A typical example of the hydrogen bond interactions observed between the ligands and the two residues is depicted in Figure 2. These residues are located near the surface-exposed loop close to the D-mannose binding pocket. Munera et al. [47] show that this region plays a role in the adhesion of UPEC strains to the uroepithelium. The inhibitors tested showed strong binding to the active site of the proteins, due to the formation of hydrogen bonds, which in turn provide stability to the protein–ligand complexes tested [48].

Our results suggest that the strongest ligand binders tested on the FimH proteins could inhibit the union of this fimbriae with the receptors of the uroepithelial cells of the bladder, making them good candidates for the development of new non-antibiotic therapeutic alternatives to counteract these UTIs. This contribution was limited by the inclusion of a relatively small number in the UPEC isolate analyzed. However, we identified and reported new molecular epidemiological data for UPEC strains that are especially valuable for understanding the epidemiology of these pathogens in Colombia. To the best of our knowledge, this is the first report from Colombia to apply *fimH* genotyping to UPEC isolates that could serve as a relatively simple sequence-based screening test, and it could be applied to a large number of UPEC strains for the characterization of recent epidemiological events and in the future. Nonetheless, we hope to contribute to molecular epidemiological studies using comprehensive and reliable methods in the future.

## 3. Conclusions

The phylogroup assignment of the UPEC strains collected for this study revealed that phylogroups D and B2 were the most prevalent and were associated with diseases outside the gastrointestinal tract. We also observed a high prevalence of the virulence genes *fimH*, *fyuA*, *chuA*, and *kpsMTII,* and found that they can be useful as molecular predictors of uropathogenic strains based on their distribution across the phylogroups and biofilm production abilities. Likewise, a high percentage of bacterial resistance of pheno-genotypic origin was present in the strains evaluated, resulting in a high rate of antibiotic therapy failure in patients. For the in silico part of this work, we decided to focus on the FimH 4XO9 and 4X5P proteins due to their broad prevalence across the *E. coli* strains isolated in this study. Based on the coupling and molecular dynamics simulations conducted, and the structural interactions and binding affinities derived from these, we proposed six ligands (b, f, g and e, i, j for 4XO9 and 4X5P, respectively) as a new non-antibiotic treatment against UPEC. These molecules could counteract the bacterial load associated with the disease, and could be evaluated in vitro and in vivo in future studies as treatment alternatives for counteracting uropathogenic infections caused by UPEC strains.

## 4. Materials and Methods

### 4.1. Bacterial Strains, Genotypic Confirmation, and Phylogenetic Analysis

One hundred and two non-duplicate uropathogenic bacterial clinical isolates of *Escherichia coli* were collected from the urine of adult patients not hospitalized during an outpatient visit at the Hospital de Cartagena, Colombia, for one year between 2018 and 2019. Patients were considered to have a UTI if the growth of a single pathogen of >10^6^ CFU per milliliter urine was observed. UPEC isolates were identified by standard methodology taking into account the criteria of the Institute of clinical and laboratory standards (CLSI 2017) [49]. The isolates were kept in brain heart infusion broth (BHI) prior to testing.

The bacterial species identification was confirmed by the presence of the *uidA* gene encoding beta-D-glucuronidase, according to Gomez et al. [50]. Subsequently, strains were assigned to one of the eight phylogenetic groups (A, B1, B2, C, D, E, F) of *E. coli sensu stricto* corresponding to *Escherichia cryptic clade*, according to Clermont et al. [51]. All primer sequences and amplified products for the target genes are described in Supplementary Table S21. Genomic DNA was extracted using the GeneJET Genomic DNA Purification Kit, Thermo Fisher Scientific, Waltham, MA, USA, according to the manufacturer′s instructions. All the PCR reactions were carried out in a total reaction volume of 25 μL containing 50 ng of template DNA, 10 μM of each primer, Dreamtaq PCR Master Mix (2) (Thermo Fisher Scientific, Waltham, MA, USA), and water. PCR products were separated on 1.5% agarose gels stained with ethidium bromide (0.5 mg/mL) and visualized with UV light.

### 4.2. Virulence Factors

#### 4.2.1. Virulence Genes

PCR was used to identify the presence of 6 virulence genes in the UPEC isolates according to the protocols described by Johnson and Stell [52] and Nakano et al. [53] (Supplementary Materials Table S22): type I fimbriae (*fimH*), yersinia-associated siderophore system (*fyuA*), marker for pathogenicity-associated island (*PAI*), uropathogenic-specific protein (*usp*), P fimbriae major and minor structural subunits (*papAH*), and group II capsular polysaccharide synthesis (*kpsMTII*).

#### 4.2.2. Biofilm-Forming Ability

The biofilm-forming ability of the isolates was screened in Congo red agar according to the protocol described by Freeman et al. [54]. Bacterial strains were seeded by depletion and incubated aerobically for 24 to 48 h at 37 °C. All plates were visually examined and the morphotypes were categorized as rdar, bdar, pdar, and saw, indicating expression of curli fimbriae and/or cellulose [55]. The quantification of Biofilm was carried out in 96-well microtiter plates using the crystal violet staining method with minor modifications [56]. Bacteria in log phase of growth (0.5 OD McFarland standards, 600 nm) were inoculated in 100 μL fresh Lysogeny Broth (LB) and incubated at 37 °C for 24 h. After incubation, the plates were washed 3 times with sterile deionized water and the adherent bacteria cells were stained with 0.5% crystal violet for 30 min. Then, the plates were washed off and solubilized with 80% ethanol and 20% acetone and were kept for 15 min. The Optical Density (OD) values of each well were measured at 492 nm. All assays were performed in triplicate in order to verify the reproducibility. Finally, the strains were classified into non-biofilm producer (OD ≤ ODc (Optical Density control)), weak biofilm producer (OD > ODc, but ≤2x ODc), moderate biofilm producer (OD > 2x ODc, but ≤4x ODc), and strong biofilm producer (OD > 4x ODc), according to the criteria of Stepanovic et al. [57]. The biofilm producer *K. pneumoniae* ATCC 700603 strain and the non-biofilm producer *E. coli* ATCC 25922 were used as controls.

### 4.3. Antibiotic Susceptibility Testing and Detection of ESBL

#### 4.3.1. Antibiotic Susceptibility Testing

Bacterial susceptibility to antibiotics was determined by the disk diffusion method according to CLSI, 2017 guidelines [49]. The following antibiotics were used: Aminoglycosides: Amikacin (AMK), Gentamicin (GEN), Tobramycin (TOB). Nitrofurans: Nitrofurantoin (NIT). Sulfonamides: Trimethoprim/sulfamethoxazole (SXT). Quinolones: Ciprofloxacin (CIP). Cephalosporins: Cefazolin (CEZ), Cefotaxime (CTX), Cefepime (FEP), Ceftazidime (CAZ), Cefoxitin (FOX), Ceftriaxone (CRO). Penicillins: Ampicillin (AM), Piperacillin (PIP), Ampicillin/sulbactam (SAM), Piperacillin/tazobactam (TZP). Carbapenems: Aztreonam (AZM), Meropenem (MEM), Ertapenem (ETP), Doripenem (DOR). Multidrug resistance (MDR) was defined as resistance (non-susceptibility) to at least one agent in three or more antimicrobial categories [58]. The MAR index was calculated according to Lazameta et al. [59] to compare the resistance level of isolates.

#### 4.3.2. Detection of ESBL

All isolates were subjected to screening for their production of Extended Spectrum beta-lactamase (ESBL) by the disk diffusion test (Kirby-Bauer disk diffusion method). Three oxyimino-cephalosporins, ceftriaxone (30 μg), ceftazidime (30 μg), and cefotaxime (30 μg), and the monobactam aztreonam (30 μg) were applied on Muller-Hinton agar plates. An inhibition zone of ≤17 mm ceftazidime, ≤22 mm cefotaxime, ≤19 mm ceftriaxone, and ≤17 mm aztreonam indicated a probable ESBL-producing strain. *Klebsiella pneumoniae* ATCC 700603 and *Escherichia coli* ATCC 25922 were used as controls and processed in the same way as isolated colonies. The double-disc synergy test (DDST) was used to confirm ESBL production. A lawn culture on a Mueller-Hinton agar plate was inoculated using a disc of Amoxicillin-Clavulanate (20/10 μg) with four cephalosporins: cefotaxime (30 μg), ceftazidime (30 μg), aztreonam (30 μg), or cefepime (30 μg), following the recommendations by CLSI [49]. A visible distortion or extension of the edge of the inhibition zone of cephalosporin towards amoxicillin/clavulanate was interpreted as positive for the production of ESBLs [59].

The existence of TEM, blaCTX-M-1, SHV, and OXA genes was determined by PCR analysis according to Oliver et al. [60] and Conceicao et al. [61] (Supplementary Materials Table S23). The PCR was performed as described elsewhere (see Section 4.1). An amplified fragment (base pairs) corresponding to 867, 876, 867, and 885 respectively, confirmed the presence of each ESBL gene.

### 4.4. Statistical Analysis

The susceptibility, phenotypes, resistance genes, virulence genes, phylogenetic assignment, and the biofilm-forming capacity present in the isolates studied were analyzed using descriptive statistics, such as frequencies and contingency tables reporting the percentages in each of the cases. The associations between the data obtained from antibiotic resistance, virulence factors, biofilm formation, and phylogenetic groups were performed using chi-square tests and Fisher’s tests. All data were initially registered in Microsoft^®^ Excel and processed with the Prism 7.02 GraphPad statistical package.

### 4.5. In Silico Phase: Evaluation of the Previously Characterized UPEC Isolates

For the in silico assays, 15 isolates were selected taking into account the previously evaluated genotypic characteristics, virulence factors, resistance to antibiotics, and production of ESBL.

#### 4.5.1. Molecular Sequences of the *fimH* Gene in the UPEC Strains

The *fimH* gene was sequenced (automatic sequencer ABI PRISM 3500) using the primers fimHF: 5′TGCAGAACGGATAAGCCGTGG′3 and fimHR: 5′GCAGTCACCTGCCCTCCGGTA′3. The homology of the *fimH* gene sequences was analyzed using GenBank and the ClustalW program [62].

#### 4.5.2. Obtaining Clean Sequences and Protein Analysis

The sequences of the *fimH* gene were translated from nucleotides to proteins, using the Blastx server (https://blast.ncbi.nlm.nih.gov/Blast.cgi, accessed on 23 March 2021). The protein sequences were compared to those reported in the NCBI database taking into account a percentage of identity in the range of 97–100% and the most representative Bit score [63]. After the verification and confirmation of the protein sequences, a crystallographic structure search in the Protein Data Bank (PDB) server was performed. Two protein structures (Protein 1: code 4X5P and Protein 2: code 4XO9) were chosen, which presented the highest prevalence within the isolates. The highest resolution crystallographic structures were chosen to perform the in silico tests in this study.

### 4.6. Inhibitor Molecule Design (Ligand Assay)

**Figure 3:**
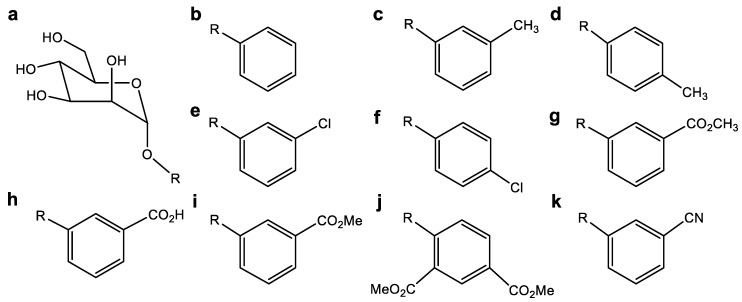
(**a**) Central nucleus of inhibitory molecules (mannose derivatives). (**b**–**k**) R substituents used as binders in the molecular coupling assays.

The molecules employed in this study were reported by Mydock-McGrane et al. [64]. This set of molecules consists of a series of inhibitors with in vitro activity for urinary infections. These molecules have shown inhibitory activity on type I fimbriae in the bacterial strains studied. In addition, after testing with the different FimH inhibitors, a hemagglutination inhibition (HAI) greater than 90% was observed, with some exceptions. The HAI data correlated well with the binding affinity of the inhibitor compounds for FimH [40,65]; therefore, inhibitors with the highest degree of affinity were chosen for the development of this work. The central nucleus of the inhibitory molecules corresponds to a mannose derivative with different R substituents (Figure 3). All molecular geometries were optimized using the 6–311G basis set together with the B3LYP functional [66,67].

### 4.7. Molecular Docking

The crystallographic structures of the two selected FimH proteins (4X5P and 4XO9) were downloaded from the PDB server and prepared using the AutoDock Vina (AutoDock 4.2) software, exploring the different interactions with the native site and/or any site (blind docking) in which the binders had greater affinity. An exhaustive search of 1000 conformations was carried out. The complexes were visualized and prepared using the software Maestro, including the addition of hydrogen atoms, assignment of binding orders, addition of partial atomic charges (using the model of Gasteiger), and elimination of water molecules. The protonation of the complex was adjusted to a physiological pH of 5.0 to simulate the urinary tract’s slight acidity [68].

The conformation of the protein did not change during the coupling procedure and the flexibility of the binders was taken into account by allowing the rotation around the flexible torsion angles of the protein. The potential maps were calculated using the Autogrid4 package included in the AutoDock program, using a dark three-dimensional box that encloses the proteins. Protein 1 (code PDB 4 × 5P) was centered at x = 1.022, y = 51.956, and z = −30.39, and had a size of x = 44 Å, y = 46 Å, and z = 40 Å, while protein 2 (code PDB 4XO9) was centered at x = 21.084, y = 13.308, and z = 33.523, and had a size of x = 84 Å, y = 66 Å, and z = 120 Å. The coupling energies were calculated as the sum of the intermolecular interaction energy and the internal energy of the binder. The couplings were classified according to their coupling energies resulting from the search of the best interaction energy using the genetic algorithm of Lamarckian order to obtain the best interaction of proteins with inhibitors [69,70,71].

### 4.8. Molecular Dynamic Simulation

Molecular dynamics simulations were performed using the AMBER18 package. The tleap program was employed to build the complexes [72,73]. The ligand within the protein–ligand complex was removed from the native crystalline structure before performing the simulation. The ff14SB force-field was employed for the amino acids’ parameters, and the Antechamber program [74] was employed to build the topology and parameters of the ligands. Every complex was solvated in a water box with TIP4PEW parameters. An initial molecular mechanics minimization of 1000 steps using the most pronounced descent algorithm followed by 1000 steps of conjugate gradients were carried out. The minimization system was heated in two stages, the first from 0 to 250 K and the second from 250 to 300 K. The production stage was performed during 200 ns for each of the systems in an isothermal-isobaric assembly (NPT) at 300 K. Snapshots were collected with a frequency of 200 ps, obtaining 1000 poses as a final result in each simulation process. All molecular dynamics simulations were carried out with a time-step of 2 fs. The free energy calculation was performed using two methodologies, the Molecular Mechanics/Poisson–Boltzmann Surface Area (MM-PBSA) and Molecular Mechanics/Generalized Born Surface Area (MM-GBSA) [75,76].

## Figures and Tables

**Table 1:** Prevalence of virulence factors in various phylogenetic groups of isolated UPEC.

Phylogroup	A (14.7%) *n* = 15	B1 (5%) *n* = 6	B2 (23.5%) *n* = 24	C (9.8%) *n* = 10	D (26.4%) *n* = 27	E (0.98%) *n* = 1	F (3.9%) *n* = 4	Clade I (7.8%) *n* = 8	N/D (6.8%) *n* = 7
Characteristics									
Biofilm									
Non-adherent	0 (0%)	1 (16.6%)	0 (0%)	0 (0%)	1 (3%)	1 (100%)	0 (0%)	1 (12.5%)	1 (14.2%)
Weak	9 (60%)	3 (50%%)	7 (29.1%)	5 (50%)	10 (37%)	0 (0%)	1 (25%)	2 (25%)	3 (42.8%)
Moderate	6 (40%)	2 (33.3%)	10 (41.6%)	5 (50%)	13 (48.1%)	0 (0%)	2 (50%)	5 (62.5%)	3 (42.8%)
Strong	0 (0%)	0 (0%)	7 (29.1%)	0 (0%)	3 (11.1%)	0 (0%)	1 (25%)	0 (0%)	0 (0%)
Pathogenicity									
pdar	5 (33.3%)	3 (50%)	0 (0%)	4 (40%)	16 (59.2%)	0 (0%)	0 (0%)	2 (25%)	0 (0%)
rdar	6 (40%)	0 (0%)	4 (16.6%)	3 (30%)	1 (3.7%)	0 (0%)	1 (25%)	2 (25%)	5 (71.4%)
bdar	4 (26.6%)	3 (50%)	20 (83.3%)	3 (30%)	10 (37%)	1 (100%)	3 (75%)	4 (50%)	2 (28.5%)
Virulence Factors									
*fimH*	14 (93.3%)	6 (100%)	24 (100%)	10 (100%)	26 (96.2%)	0 (0%)	4 (100%)	8 (100%)	5 (71.4%)
*fyuA*	11 (73.3%)	4 (66.6%)	22 (91.6%)	7 (70%)	24 (88.8%)	1 (100%)	4 (100%)	7 (87.5%)	6 (85.7%)
*PAI*	2 (13.3%)	1 (16.6%)	11 (45.8%)	6 (60%)	7 (25.9%)	0 (0%)	1 (25%)	2 (25%)	2 (28.5%)
*Usp*	0 (0%)	0 (0%)	10 (41.6%)	0 (0%)	2 (7.4%)	0 (0%)	1 (25%)	0 (0%)	2 (28.5%)
*papAH*	2 (13.3%)	0 (0%)	9 (37.5%)	5 (50%)	9 (33.3%)	0 (0%)	2 (50%)	2 (25%)	2 (28.5%)
*kpsMTII*	5 (33.3%)	1 (16.6%)	23 (95.8%)	6 (60%)	23 (85.1)	0 (0%)	4 (100%)	3 (37.5%)	4 (57.1%)

**Table 2:** Antimicrobial susceptibility pattern of isolated UPEC.

Antibiotics (Disk Concentration)	% R	% I	% S
Aminoglycosides			
Amikacin (AMK) (30 µg)	8.4	-	91.6
Gentamicin (GEN) (10 µg)	27.4	-	72.6
Tobramycin (TOB) (10 µg)	24.2	13.2	62.6
Cephalosporins			
Cefazolin (CFZ) (30 µg)	45.8	5.8	48.4
Cefepime (FEP) (30 µg)	54.2	-	45.8
Cefotaxime (CTX) (30 µg)	44.2	-	55.8
Cefoxitin (FOX) (30 µg)	6.3	4.2	89.5
Ceftazidime (CAZ) (30 µg)	11.1	-	88.9
Ceftriaxone (CAX) (30 µg)	5.3	3.7	91
Quinolones			
Ciprofloxacin (CIP) (5 µg)	49.5	-	50.5
Carbapenems			
Doripenem (DOR) (8 µg)	4.2	-	95.8
Ertapenem (ETP) (4 µg)	4.2	-	95.8
Aztreonam (ATM) (30 µg)	33	13	54
Meropenem (MEM) (10 µg)	9.5	-	90.5
Nitrofurans			
Nitrofurantoin (NIT) (300 µg)	11.1	-	88.9
Penicillins			
Ampicillin (AMP) (10 µg)	88.4	-	12.6
Pip/tazo (TZP) (100/10 µg)	5.8	7.9	86.3
Piperacillin (PIP) (100 µg)	45.2	-	51.1
Amp/Sulbactam (SAM) (10 µg)	42.6	19	38.4
Sulfonamides			
Trimet/sulfa (SXT) (0.25/23.7 µg)	50.5	-	49.5

**Table 3:** Genotypic characteristics, virulence factors, resistance to antibiotics, and production of ESBL of UPEC selected.

Strains	Phylogenetic Group	Virulence Factors	Biofilm Forming Ability	Resistance to Antibiotics	
			Morphotypes/ Type of Biofilm	Antibiotic Susceptibility	*MDR/* *ESBL* Genes
AMR1638	A	*fyuA, fimH*	rdar/Moderate	AMP	-/-
AMR1676	B1	*fimH*	bdar/weak	-	-/-
AMR4129	B1	*fyuA, kpsMTII, fimH*	bdar/non-producer	AMK; GEN; TOB; SXT; CIP; CEZ; FEP; CAZ; FOX; CRO; AMP; PIP; SAM; AZM.	+/-
AMR1201	B2	*fyuA, kpsMTII, usp, fimH*	bdar/Moderate	GEN; TOB; NIT; SXT; CIP; CEZ; CTX; FEP; CAZ; FOX; CRO; AMP; PIP; * SAM.	+/SHV
AMR4620	B2	*fyuA, kpsMTII, usp, PAI, fimH*	bdar/strong	GEN; TOB; SXT; CIP; CTX; FEP; CAZ; FOX; CRO; AMP; PIP; SAM; TZP; AZM.	+/OXA
AMR1898	B2	*fyuA, kpsMTII, usp, papAH, fimH*	bdar/Moderate	-	-/SHV
AMR1740	B2	*fyuA, kpsMTII, papAH, fimH*	bdar/strong	GEN; TOB; * NIT; SXT; CTX; FEP; CAZ; FOX; CRO; AMP; PIP; SAM; AZM.	+/-
AMR2919	B2	*fyuA, kpsMTII, usp, papAH, fimH*	bdar/non-producer	GEN; * TOB; NIT; SXT; CIP; CEZ; CTX; FEP; CAZ; CRO; AMP; PIP; * SAM	+/-
AMR3633	C	*fimH*	bdar/weak	-	-/CTX-M-1
AMR1598	Clade I	*fyuA, kpsMTII, papAH, fimH*	rdar/Moderate	GEN; TOB; * NIT; SXT; CIP; CTX; FEP; CAZ; FOX; CRO; AMP; PIP; SAM; * TZP; * AZM	+/TEM
AMR4671	D	*fyuA, papAH, fimH*	pdar/Moderate	-	-/-
AMR0843	D	*fimH*	rdar/Moderate	-	-/TEM
AMR1642	D	*fuyA, fimH*	bdar/Moderate	GEN; TOB; SXT; CIP; CEZ; CTX; FEP; CAZ; CRO; AMP; PIP; SAM.	+/OXA
AMR0864	F	*fyuA, kpsMTII, fimH*	rdar/strong	GEN; * TOB; CEZ; CTX; FEP; CAZ; CRO; AMP; PIP; SAM.	+/-
AMR3525	F	*fyuA, kpsMTII, fimH*	bdar/weak	AMK; GEN; TOB; NIT; SXT; CIP; CEZ; CTX; FEP; CAZ; FOX; CRO; AMP; PIP; SAM; TZP; AZM.	+/-

Positive +; Negative -; * Intermediate resistance; Amikacin: AMK; Gentamicin: GEN; Tobramycin: TOB; Nitrofurantoin: NIT; Trimethoprim/sulfamethoxazole: SXT; Ciprofloxacin: CIP; Cefazolin: CEZ; Cefotaxime: CTX; Cefepime: FEP; Ceftazidime: CAZ; Cefoxitin: FOX; Ceftriaxone: CRO; Ampicillin: AMP; Piperacillin: PIP; Ampicillin/sulbactam: SAM; Piperacillin/tazobactam: TZP; Aztreonam: AZM; Meropenem: MEM; Ertapenem: ETP; Doripenem: DOR.

**Table 4:** Calculated affinities and RMSDs for the most stable conformation of each binder within each protein.

Protein 4X5P				Protein 4XO9		
Ligand	Affinity (kcal/mol)	Distance RMSD (Å)	Mode RMSD (Å)	Affinity (kcal/mol)	Distance RMSD (Å)	Mode RMSD (Å)
Native	−6.6	0.097	1.899	−6.6	0.080	2.122
b	−6.7	0.462	1.215	−6.6	0.050	1.144
c	−6.8	0.371	1.900	−6.9	0.570	1.554
d	−7.0	0.096	1.111	−6.9	1.826	2.232
e	−6.5	0.080	1.117	−6.7	0.063	1.109
f	−6.8	1.437	1.750	−6.6	0.159	1.125
g	−6.5	0.094	1.625	−6.8	0.527	1.137
h	−6.7	0.166	1.111	−6.9	2.228	3.856
i	−6.6	0.525	1.343	−6.8	0.257	1.064
j	−6.5	0.366	3.804	−6.7	0.293	3.726
k	−6.5	0.370	1.483	−7.0	0.649	1.133

**Table 5:** Free binding energy to the 4XO9 protein calculated using MM/GBSA and MM/PBSA methods.

MM/GBSA				MM/PBSA		
Ligand	Energy Average	Standard Deviation	Standard Error	Energy Average	Standard Deviation	Standard Error
Native	−20.5170	3.1661	0.1837	−2.2483	4.0371	0.3291
b	−40.0576	5.6342	0.1783	−22.0351	5.4900	0.1737
c	−19.8246	7.2451	1.7077	6.4182	9.509	2.2413
d	−9.0984	5.9672	0.1888	−0.2142	4.6101	0.1484
e	−9.6600	6.1696	0.1952	0.1840	4.4867	0.1420
f	−20.4026	6.3241	0.2001	−2.3836	6.4959	0.2055
g	−27.5406	7.8822	0.2494	−8.2752	11.2063	0.3546
h	−18.8009	7.5942	0.2403	−2.7163	6.9245	0.2191
i	−18.9992	3.5661	0.1128	−2.3183	5.6019	0.1772
j	−7.8151	5.9003	0.1867	0.3845	3.2391	0.1022
k	−10.7836	6.7607	0.2139	−0.9951	6.0892	0.1927

**Table 6:** Free binding energy to the 4X5P protein calculated using MM/GBSA and MM/PBSA methods.

MM/GBSA				MM/PBSA		
Ligand	Energy Average	Standard Deviation	Standard Error	Energy Average	Standard Deviation	Standard Error
Native	−42.3971	4.1549	0.1171	−21.6338	5.1121	0.1732
b	−41.1313	4.7338	0.1498	−24.6143	4.5592	0.1442
c	−43.0973	3.8352	0.1213	−23.7164	4.8470	0.1534
d	−40.2022	5.1058	0.1615	−22.4473	5.5330	0.1750
e	−45.9053	3.6061	0.1141	−28.1108	3.5312	0.1170
f	−44.3710	4.5614	0.1443	−26.7404	3.9121	0.1238
g	−42.7031	4.8568	0.1537	−22.9272	5.1634	0.1634
h	−42.5463	4.6572	0.1473	−25.2265	4.4345	0.1403
i	−45.0402	3.8545	0.1220	−27.0876	3.7791	0.1196
j	−46.5859	4.0885	0.1294	−24.6417	4.7458	0.1502
k	−44.4012	4.1211	0.1304	−24.3189	5.0090	0.1585

**Table 7:** Percentage of simulation time when the hydrogen bonds with ASP 54 and GLN 133 were formed for different ligands.

Protein 4XO9		
Ligand	GLN 133	ASP 54
b	71.30%	68.20%
f	-	54.30%
g	98.90%	99.90%
Protein 4X5P		
e	99.90%	100%
i	99.90%	99.80%
j	-	99.90%

## Supplementary Materials

The following are available online at https://www.mdpi.com/article/10.3390/antibiotics10091072/s1, Tables S1–S3 detail the primer sequences used in this study. Molecular coupling results from AutoDock Vina obtained for the 4X5P and 4XO9 proteins with each of the ligands are presented in Tables S4–S23. Figures S1–S22 show the root-mean-square deviation (RMSD) along the molecular dynamic simulations.
