# Supplementary material for: D-Mannoside FimH Inhibitors as Non-Antibiotic Alternatives for Uropathogenic Escherichia coli
Source: Antibiotics (Basel). 2021 Sep 4;10(9):1072. doi: 10.3390/antibiotics10091072 (PMC8465801; doi:10.3390/antibiotics10091072)
Supplement: Supplementary file 1 [file antibiotics-10-01072-s001.zip › antibiotics-1309867-supplementary.pdf]

Supplementary Materials

D- Mannoside FimH inhibitors as non-antibiotic alternatives for Uropathogenic *Escherichia coli*

Alfredo Montes-Robledo [1,2], Rosa Baldiris-Avila[1,2,3,*] and Johan Fabian Galindo[4,*]

[1] Grupo de Investigación Microbiología Clínica y Ambiental, Facultad de Ciencias Exactas y Naturales, Universidad de Cartagena, Cartagena de Indias, Colombia amontesr@unicartagena.edu.co

[2] Maestría en Microbiología, Facultad de Medicina, Universidad de Cartagena, Cartagena de Indias, Colombia. rbaldirisa@unicartagena.edu.co

[3] Grupo de Investigación CIPTEC, Facultad de Ingeniería, Programa de Ingeniería de Procesos, Fundación Universitaria Tecnológico Comfenalco, Cartagena.

[4] Departamento de Quı́mica, Universidad Nacional de Colombia, Bogota, Colombia. jfgalindoc@unal.edu.co

***** Correspondence: authors information:

Rosa Baldiris Avila, Facultad de Ciencias Exactas y Naturales, Programa de Biología, Universidad de Cartagena. **E**-mail: rbaldirisa@unicartagena.edu.co

Johan Fabian Galindo, Departamento de Química, Universidad Nacional de Colombia, Bogotá, Colombia. E-mail: jfgalindoc@unal.edu.co

Alfredo Montes Robledo, Facultad de Ciencias Exactas y Naturales, Programa de Biología, Universidad de Cartagena. **E**-mail: amontesr@unicartagena.edu.co

Table S1. Molecular coupling results obtained from the 4X5P code protein with the ligand b made in the AutoDock Vina software package.

Protein 4X5P			
Positions Ligand b	Afinity (kcal/ mol)	Distance RMSD (Å)	Mode RMSD (Å)
**1**	-6.7	0.000	0.000
**2**	-6.7	0.462	1.215
**3**	-6.6	1.334	2.107
**4**	-6.6	1.346	1.847
**5**	-6.4	1.958	2.657
**6**	-6.4	0.882	1.394
**7**	-6.2	2.064	3.256
**8**	-6.1	1.208	1.863
**9**	-6.1	1.590	2.026

Table S2. Molecular coupling results obtained from the 4X5P code protein with the ligand c made in the AutoDock Vina software package.

Protein 4X5P			
Positions Ligand c	Afinity (kcal/ mol)	Distance RMSD (Å)	Mode RMSD (Å)
**1**	-6.8	0.000	0.000
**2**	-6.8	1.102	1.900
**3**	-6.7	0.371	1.605
**4**	-6.6	1.629	2.341
**5**	-6.4	1.818	3.325
**6**	-6.4	1.171	1.971
**7**	-6.2	2.384	3.326
**8**	-6.1	1.749	3.517
**9**	-6.1	1.767	2.451

Table S3. Molecular coupling results obtained from the 4X5P code protein with the ligand d made in the AutoDock Vina software package.

Protein 4X5P			
Positions Ligand d	Afinity (kcal/ mol)	Distance RMSD (Å)	Mode RMSD (Å)
**1**	-7.0	0.000	0.000
**2**	-6.9	0.096	1.111
**3**	-6.7	1.395	2.131
**4**	-6.7	1.418	1.921
**5**	-6.4	0.893	1.013
**6**	-6.4	2.048	2.772
**7**	-6.3	2.019	2.727
**8**	-6.2	2.136	3.183
**9**	-6.2	1.783	2.456

Table S4. Molecular coupling results obtained from the 4X5P code protein with the ligand e made in the AutoDock Vina software package.

Protein 4X5P			
Positions Ligand e	Afinity (kcal/mol)	Distance RMSD (Å)	Mode RMSD (Å)
**1**	-6.8	0.000	0.000
**2**	-6.8	0.080	1.117
**3**	-6.6	1.470	2.079
**4**	-6.6	1.500	1.858
**5**	-6.4	1.193	1.213
**6**	-6.3	2.301	2.776
**7**	-6.2	2.096	3.151
**8**	-6.1	2.577	3.014
**9**	-6.1	1.526	1.761

Table S5. Molecular coupling results obtained from the 4X5P code protein with the ligand f made in the AutoDock Vina software package.

Protein 4X5P			
Positions Ligand f	Afinity (kcal/mol)	Distance RMSD (Å)	Mode RMSD (Å)
**1**	-6.5	0.000	0.000
**2**	-6.3	1.437	1.750
**3**	-6.2	2.041	2.268
**4**	-6.2	1.374	2.007
**5**	-5.9	1.897	3.476
**6**	-5.9	2.080	3.134
**7**	-5.8	3.481	2.808
**8**	-5.7	2.204	3.054
**9**	-5.5	2.704	3.481

Table S6. Molecular coupling results obtained from the 4X5P code protein with the ligand g made in the AutoDock Vina software package.

Protein 4X5P			
Positions Ligand g	Afinity (kcal/mol)	Distance RMSD (Å)	Mode RMSD (Å)
**1**	-6.5	0.000	0.000
**2**	-6.5	2.411	2.812
**3**	-6.5	1.194	1.625
**4**	-6.4	1.782	3.080
**5**	-6.4	2.435	4.035
**6**	-6.4	2.023	3.354
**7**	-6.3	3.392	5.425
**8**	-6.2	2.061	2.570
**9**	-6.2	3.317	4.444

Table S7. Molecular coupling results obtained from the 4X5P code protein with the ligand h made in the AutoDock Vina software package.

Protein 4X5P			
Positions Ligand h	Afinity (kcal/mol)	Distance RMSD (Å)	Mode RMSD (Å)
**1**	-6.7	0.000	0.000
**2**	-6.5	2.166	3.111
**3**	-6.4	1.595	1.978
**4**	-6.3	1.189	1.325
**5**	-6.3	2.863	4.398
**6**	-6.3	1.419	1.938
**7**	-6.2	2.636	3.280
**8**	-6.1	27.602	29.642
**9**	-6.1	2.019	3.138

Table S8. Molecular coupling results obtained from the 4X5P code protein with the ligand i made in the AutoDock Vina software package.

Protein 4X5P			
Positions Ligand i	Afinity (kcal/mol)	Distance RMSD (Å)	Mode RMSD (Å)
**1**	-6.6	0.000	0.000
**2**	-6.6	0.525	1.343
**3**	-6.5	1.984	2.702
**4**	-6.5	1.954	2.467
**5**	-6.1	2.705	3.685
**6**	-6.1	2.902	3.669
**7**	-6.0	1.672	2.607
**8**	-5.9	1.555	3.073
**9**	-5.9	1.554	1.765

Table S9. Molecular coupling results obtained from the 4X5P code protein with the ligand j made in the AutoDock Vina software package.

Protein 4X5P			
Positions Ligand j	Afinity (kcal/mol)	Distance RMSD (Å)	Mode RMSD (Å)
**1**	-6.5	0.000	0.000
**2**	-6.5	0.366	3.804
**3**	-6.5	1.670	2.217
**4**	-6.4	0.945	3.572
**5**	-6.4	0.971	1.121
**6**	-6.4	1.479	4.061
**7**	-6.3	2.081	4.455
**8**	-6.3	1.623	2.591
**9**	-6.2	1.288	1.538

Table S10. Molecular coupling results obtained from the 4X5P code protein with the ligand k made in the AutoDock Vina software package.

Protein 4X5P			
Positions Ligand k	Afinity (kcal/mol)	Distance RMSD (Å)	Mode RMSD (Å)
**1**	-6.7	0.000	0.000
**2**	-6.4	2.370	3.483
**3**	-6.4	1.218	1.629
**4**	-6.4	1.910	2.484
**5**	-6.3	1.599	2.240
**6**	-6.3	1.411	2.324
**7**	-6.3	2.113	2.483
**8**	-6.2	2.207	2.525
**9**	-6.0	1.908	3.561

Table S11. Molecular coupling results obtained from the 4XO9 code protein with the ligand b made in the AutoDock Vina software package.

Protein 4XO9			
Positions Ligand b	Afinity (kcal/mol)	Distance RMSD (Å)	Mode RMSD (Å)
**1**	-6.6	0.000	0.000
**2**	-6.6	0.050	1.144
**3**	-6.3	67.290	69.678
**4**	-6.0	2.167	3.980
**5**	-6.0	38.312	40.132
**6**	-5.9	1.852	5.723
**7**	-5.9	58.754	60.174
**8**	-5.9	36.749	38.576
**9**	-5.8	1.832	2.582

Table S12. Molecular coupling results obtained from the 4XO9 code protein with the ligand c made in the AutoDock Vina software package.

Protein 4XO9			
Positions Ligand c	Afinity (kcal/mol)	Distance RMSD (Å)	Mode RMSD (Å)
**1**	-6.9	0.000	0.000
**2**	-6.9	0.570	1.554
**3**	-6.5	1.390	1.995
**4**	-6.5	67.010	69.893
**5**	-6.3	2.213	3.964
**6**	-6.2	1.623	2.589
**7**	-6.2	2.017	6.040
**8**	-6.2	38.193	40.243
**9**	-6.1	38.360	40.410

Table S13. Molecular coupling results obtained from the 4XO9 code protein with the ligand d made in the AutoDock Vina software package.

**Protein 4XO9**			
Positions Ligand d	Afinity (kcal/mol)	Distance RMSD (Å)	Mode RMSD (Å)
**1**	-6.7	0.000	0.000
**2**	-6.4	1.826	2.232
**3**	-6.3	1.666	2.030
**4**	-6.2	67.289	69.356
**5**	-6.1	2.786	4.556
**6**	-6.1	2.550	6.132
**7**	-6.0	58.315	59.666
**8**	-6.0	36.846	38.540
**9**	-5.9	38.163	39.822

Table S14. Molecular coupling results obtained from the 4XO9 code protein with the ligand e made in the AutoDock Vina software package.

**Protein 4XO9**			
Positions Ligand e	Afinity (kcal/mol)	Distance RMSD (Å)	Mode RMSD (Å)
**1**	-6.9	0.000	0.000
**2**	-6.9	0.063	1.109
**3**	-6.8	0.981	1.531
**4**	-6.4	1.055	1.617
**5**	-6.4	67.657	69.915
**6**	-6.2	1.802	2.712
**7**	-6.1	38.501	40.246
**8**	-6.0	37.026	38.784
**9**	-5.9	58.652	60.016

Table S15. Molecular coupling results obtained from the 4XO9 code protein with the ligand f made in the AutoDock Vina software package.

**Protein 4XO9**			
Positions Ligand f	Afinity (kcal/mol)	Distance RMSD (Å)	Mode RMSD (Å)
**1**	-6.6	0.000	0.000
**2**	-6.6	0.159	1.125
**3**	-6.3	67.672	69.913
**4**	-6.1	1.035	1.595
**5**	-6.0	1.825	2.716
**6**	-6.0	38.537	40.228
**7**	-5.9	37.087	38.790
**8**	-5.8	58.725	60.055
**9**	-5.8	36.330	38.028

Table S16. Molecular coupling results obtained from the 4XO9 code protein with the ligand g made in the AutoDock Vina software package.

**Protein 4XO9**			
Positions Ligand g	Afinity (kcal/mol)	Distance RMSD (Å)	Mode RMSD (Å)
**1**	-6.8	0.000	0.000
**2**	-6.8	0.527	1.137
**3**	-6.6	37.106	39.006
**4**	-6.6	2.288	3.958
**5**	-6.5	67.156	70.265
**6**	-6.5	1.826	2.753
**7**	-6.5	2.389	6.714
**8**	-6.3	1.946	2.558
**9**	-6.3	66.606	69.742

Table S17. Molecular coupling results obtained from the 4XO9 code protein with the ligand h made in the AutoDock Vina software package.

**Protein 4XO9**			
Positions Ligand h	Afinity (kcal/mol)	Distance RMSD (Å)	Mode RMSD (Å)
**1**	-6.9	0.000	0.000
**2**	-6.7	2.328	6.856
**3**	-6.7	36.733	38.836
**4**	-6.7	66.856	70.107
**5**	-6.6	2.120	6.745
**6**	-6.5	2.292	6.551
**7**	-6.5	66.503	69.774
**8**	-6.5	1.710	2.410
**9**	-6.4	2.237	3.786

Table S18. Molecular coupling results obtained from the 4XO9 code protein with the ligand i made in the AutoDock Vina software package.

**Protein 4XO9**			
Positions Ligand i	Afinity (kcal/mol)	Distance RMSD (Å)	Mode RMSD (Å)
1	-6.8	0.000	0.000
2	-6.8	0.257	1.064
3	-6.8	0.767	1.122
4	-6.7	0.756	1.496
5	-6.2	1.694	2.517
6	-6.1	67.400	70.331
7	-5.9	37.168	38.872
8	-5.9	58.223	60.760
9	-5.9	38.353	40.057

Table S19. Molecular coupling results obtained from the 4XO9 code protein with the ligand j made in the AutoDock Vina software package.

**Protein 4XO9**			
Positions Ligand j	Afinity (kcal/mol)	Distance RMSD (Å)	Mode RMSD (Å)
**1**	-6.7	0.000	0.000
**2**	-6.7	0.293	3.726
**3**	-6.7	37.039	39.580
**4**	-6.6	37.027	39.782
**5**	-6.4	67.374	71.153
**6**	-6.4	67.396	71.111
**7**	-6.2	38.419	41.176
**8**	-6.2	38.324	40.951
**9**	-6.1	2.891	4.547

Table S20. Molecular coupling results obtained from the 4XO9 code protein with the ligand k made in the AutoDock Vina software package.

**Protein 4XO9**			
Positions Ligand k	Afinity (kcal/mol)	Distance RMSD (Å)	Mode RMSD (Å)
**1**	-7.0	0.000	0.000
**2**	-6.4	67.649	70.133
**3**	-6.3	2.234	3.178
**4**	-6.3	59.324	60.659
**5**	-6.3	1.655	2.281
**6**	-6.3	1.872	2.302
**7**	-6.2	1.970	2.464
**8**	-6.1	2.321	3.177
**9**	-6.1	37.274	39.418

Table S21. Primers for classification of phylogenetic groups in *Escherichia coli*.

Gene	Sequence (5´ to 3´)	Amplicon size (bp)	Optimal Annealing Temperature (ºC)	Reference
chuA	F: ATGGTACCGGACGAACCAAC	288	59	[51]
	R: TGCCGCCAGTACCAAAGACA			
yjaA	F: CAAACGTGAAGTGTCAGGAG	211		
	R: AATGCGTTCCTCAACCTGTG			
TspE4.C2	F: CACTATTCGTAAGGTCATCC	152		
	R: AGTTTATCGCTGCGGGTCGC			
arpA	F: AACGCTATTCGCCAGCTTGC	400		
	R: TCTCCCCATACCGTACGCTA			
trpA	F: AGTTTTATGCCCAGTGCGAG	219		
	R: TCTGCGCCGGTCACGCCC			
arpA	F: GATTCCATCTTGTCAAAATATGCC	301	57	
	R: GAAAAGAAAAAGAATTCCCAAGAG			
trpA	F: CGGCGATAAAGACATCTTCAC	489		
	R: GCAACGCGGCCTGGCGGAAG			

Table S22. Primers for identification of virulence factors in uropathogenic UPEC strains.

Gene	Primer sequence (5´ to 3´)	Amplicon size (bp)	Reference
KpsMT II F	GCGCATTTGCTGATACTGTTG	272	
KpsMT II R	CATCCAGACGATAAGCATGAGCA		
fimH F	TGCAGAACGGATAAGCCGTGG	508	
fimH R	GCAGTCACCTGCCCTCCGGTA		
PAI F	GGACATCCTGTTACAGCGCGCA	930	[52]
PAI R	TCGCCACCAATCACAGCCGAAC		
papAH F	ATGGCAGTGGTGTCTTTTGGTG	720	
papAH R	CGTCCCACCATACGTGCTCTTC		
fyuA F	TGATTAACCCCGCGACGGGAA	880	
fyuA R	CGCAGTAGGCACGATGTTGTA		
Usp F	ATGCTACTGTTTCCGGGTAGTGTGT	1000	[53]
Usp R	CATCATGTAGTCGGGGCGTAACAAT		

Table S23. Primers for identification of bacterial resistance genes.

Gene	Primer sequence (5´ to 3´)	Amplicon size (bp)	Reference
TEM-F	ATGAGTATTCAACATTTCCG	867	
TEM-R	CTGACAGTTACCAATGCTTA		[60]
SHV-F	GGTTATGCGTTATATTCGCC	867	
SHV-R	TTAGCGTTGCCAGTGCTC		
CTX-M-1F	ATGGTTAAAAAATCACTGCGYC	876	[61]
CTX-M-1R	TTA CAA ACC GTC GGT G		
OXA-F	ACACAATACATATCAACTTCGC	885	[60]
OXA-R	AGTGTGTTTAGAATGGTGATC		

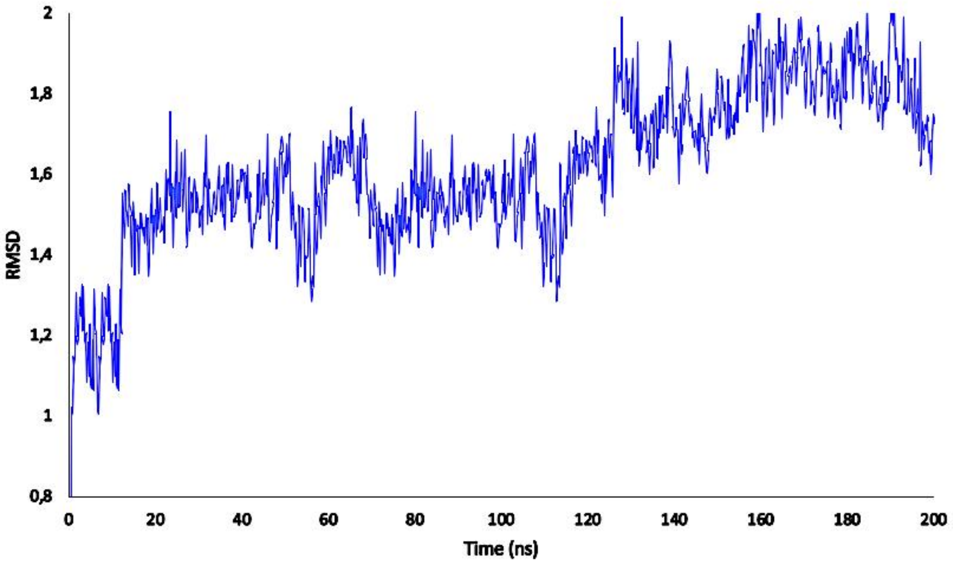

Figure S1. Visualization of root-mean-square deviation (RMSD) of the 4X5P protein with the ligand native.

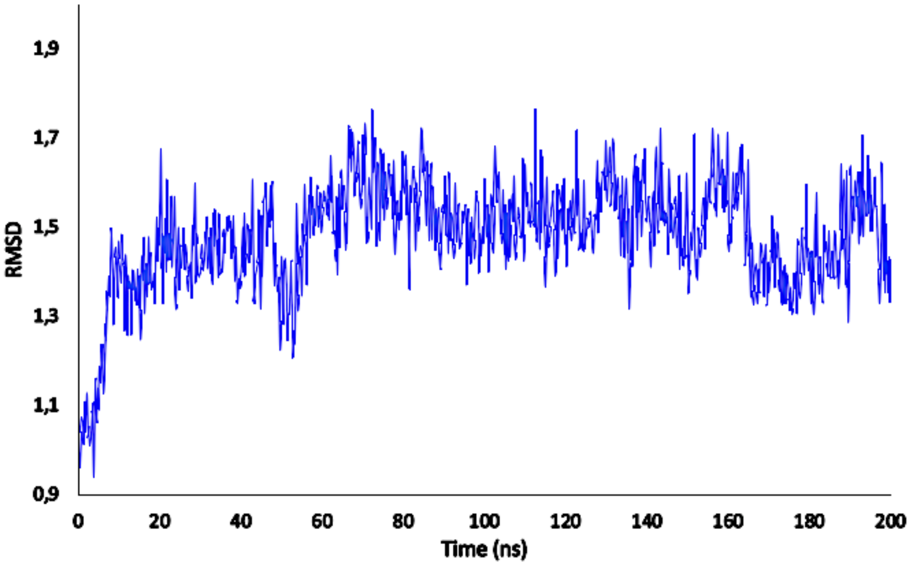

Figure S2. Visualization of root-mean-square deviation (RMSD) of the 4X5P protein with the ligand b.

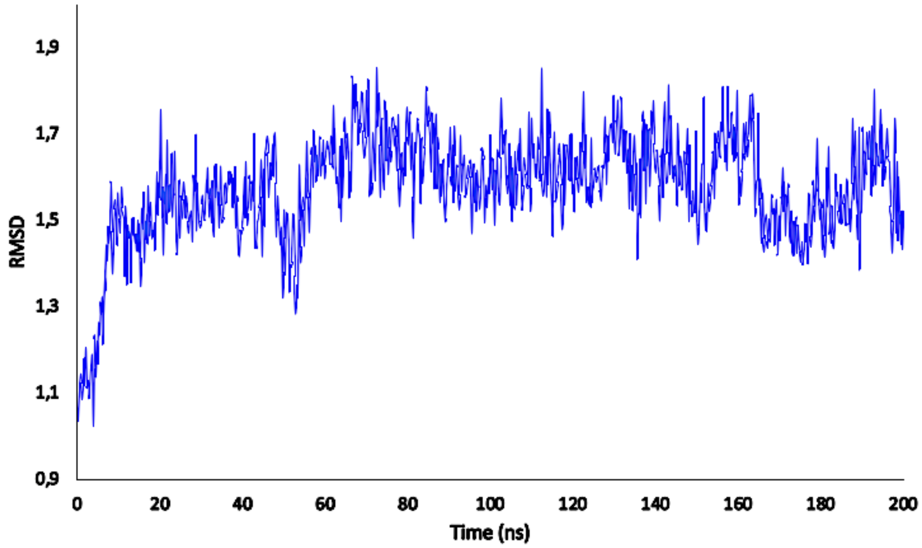

Figure S3. Visualization of root-mean-square deviation (RMSD) of the 4X5P protein with the ligand c.

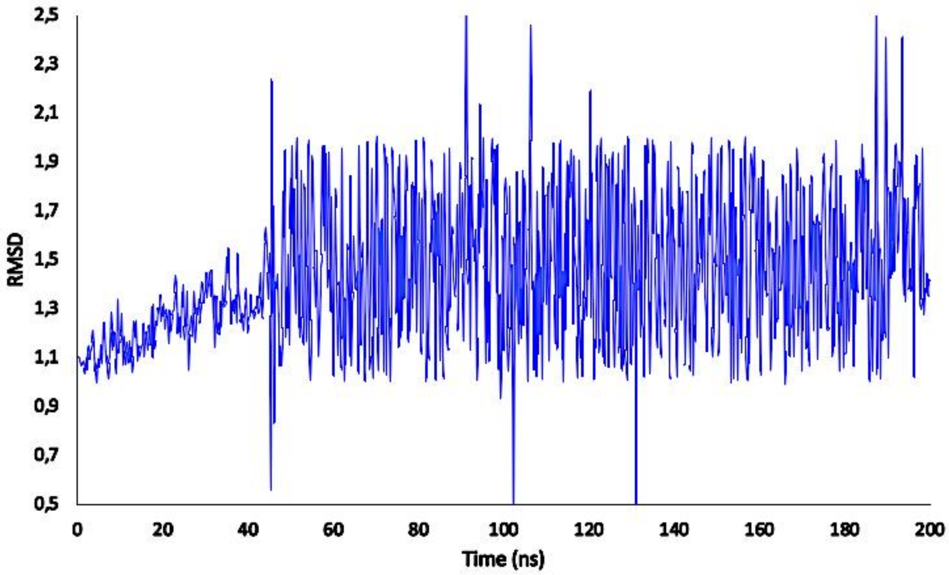

Figure S4. Visualization of root-mean-square deviation (RMSD) of the 4X5P protein with the ligand d.

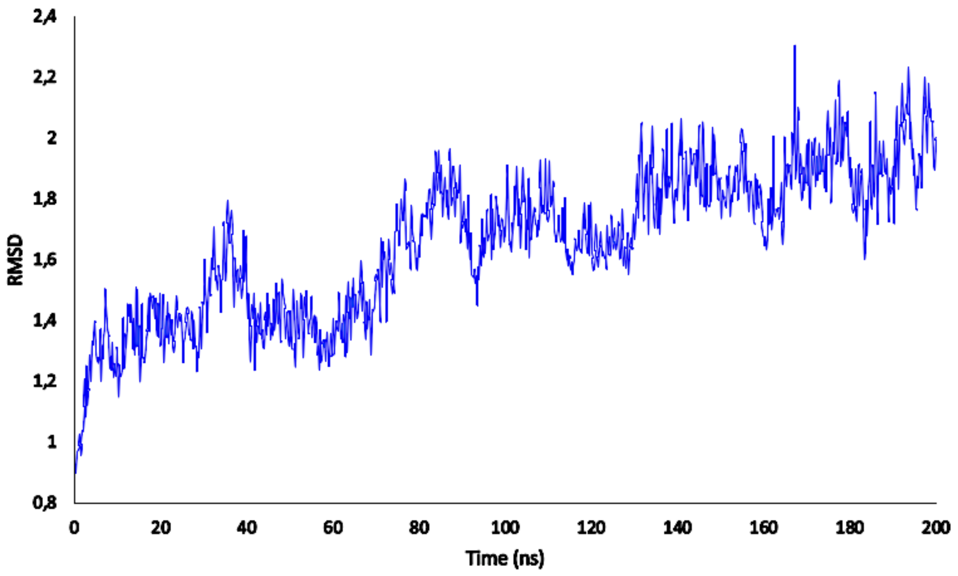

Figure S5. Visualization of root-mean-square deviation (RMSD) of the 4X5P protein with the ligand e.

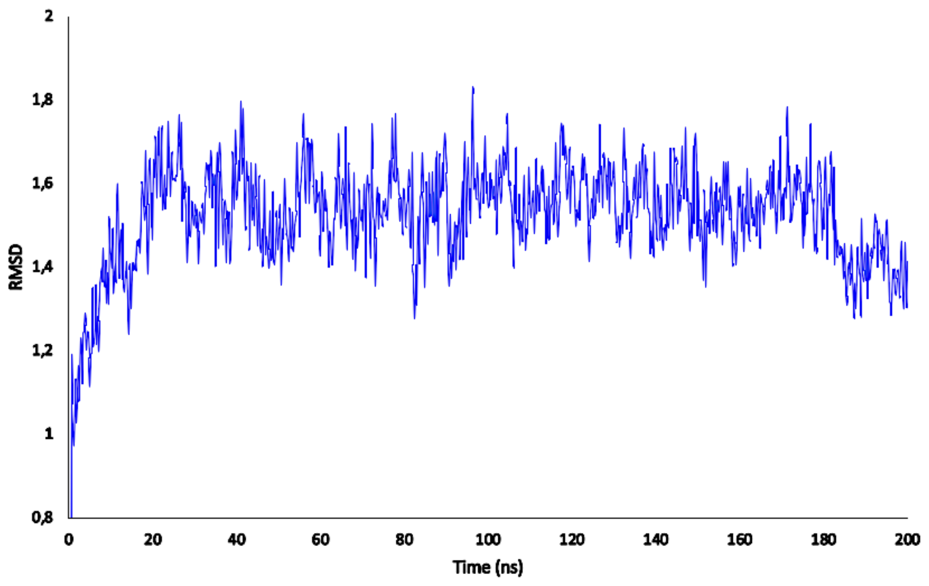

Figure S6. Visualization of root-mean-square deviation (RMSD) of the 4X5P protein with the ligand f

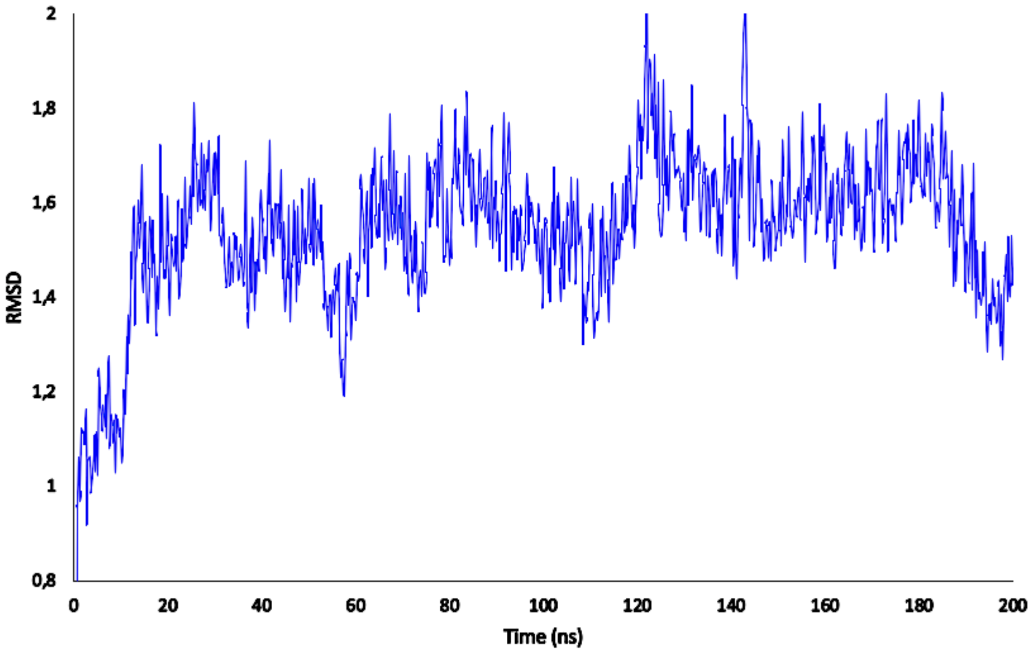

Figure S7. Visualization of root-mean-square deviation (RMSD) of the 4X5P protein with the ligand g.

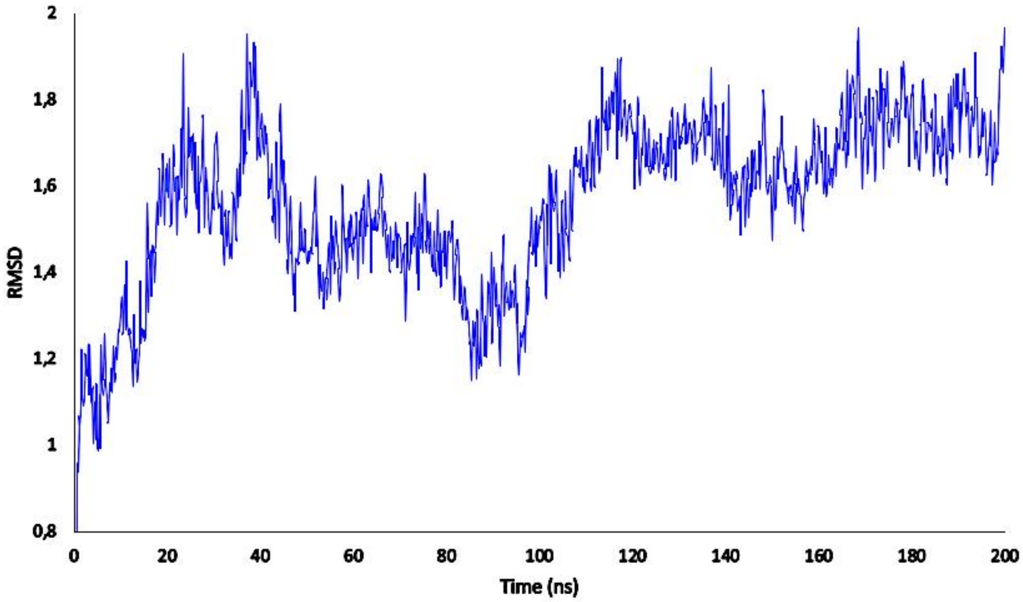

Figure S8. Visualization of root-mean-square deviation (RMSD) of the 4X5P protein with the ligand h.

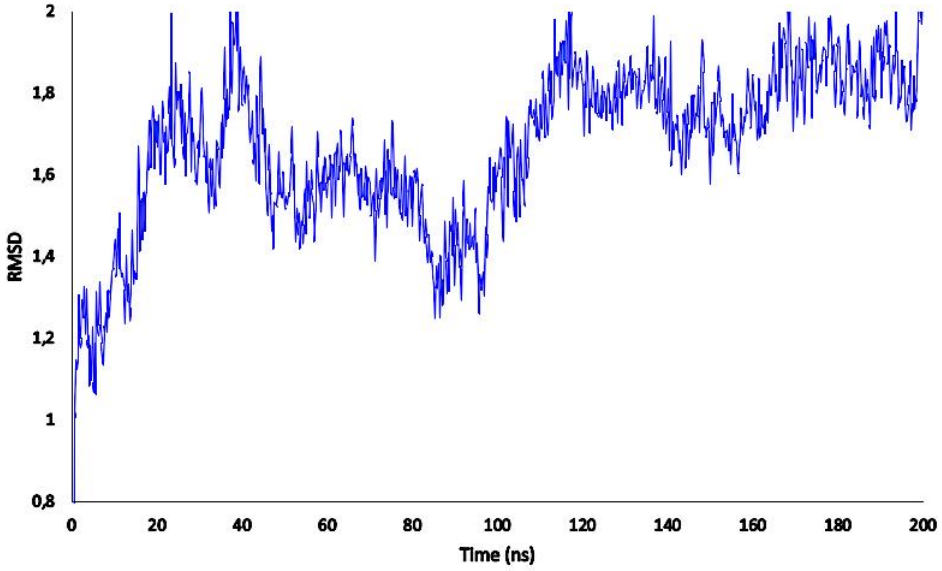

Figure S9. Visualization of root-mean-square deviation (RMSD) of the 4X5P protein with the ligand i.

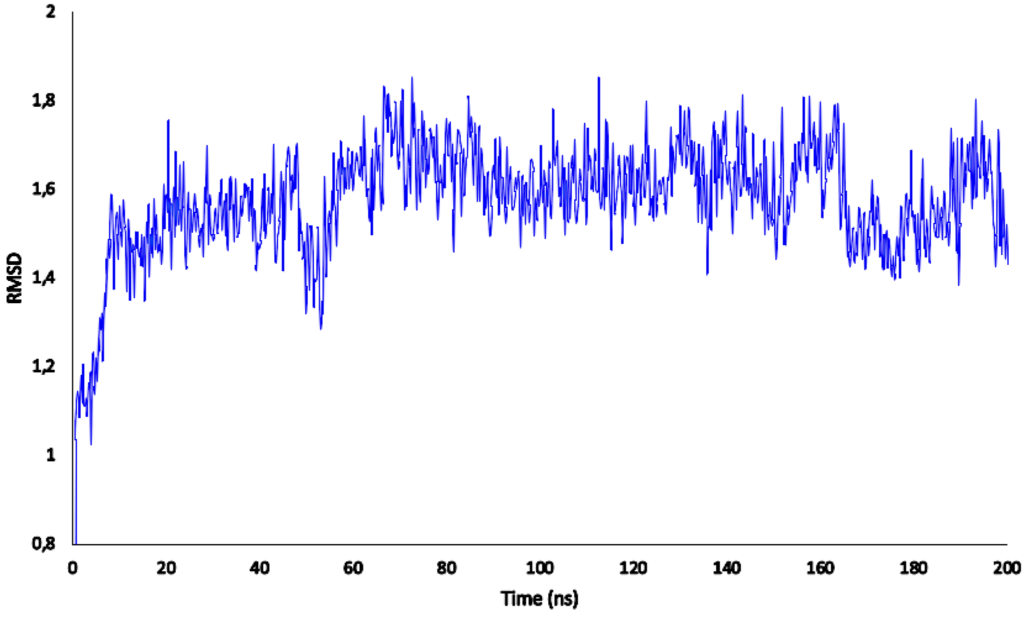

Figure S10. Visualization of root-mean-square deviation (RMSD) of the 4X5P protein with the ligand j.

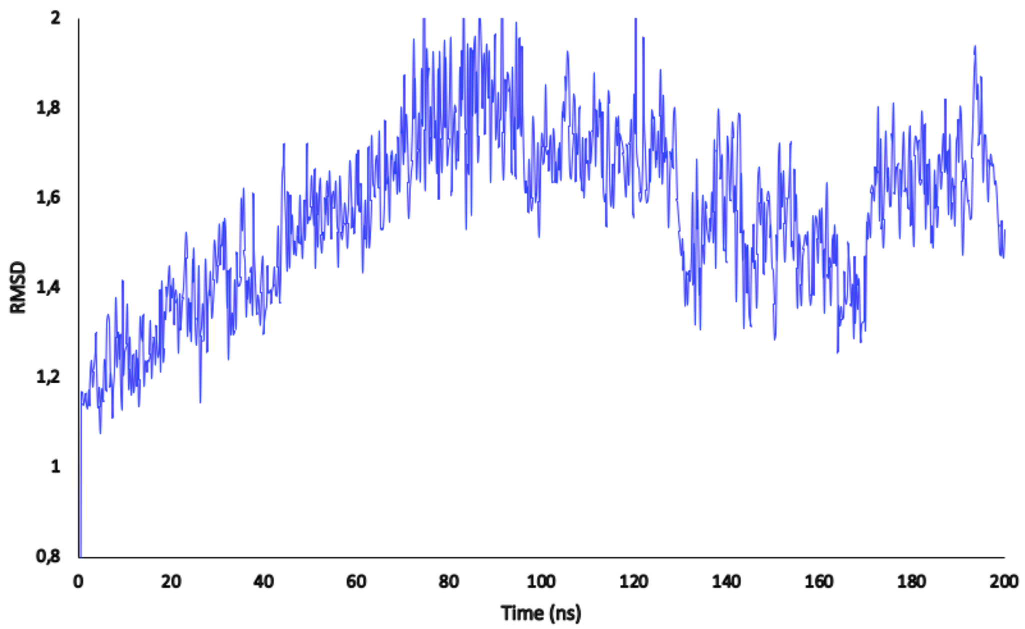

Figure S11. Visualization of root-mean-square deviation (RMSD) of the 4X5P protein with the ligand k.

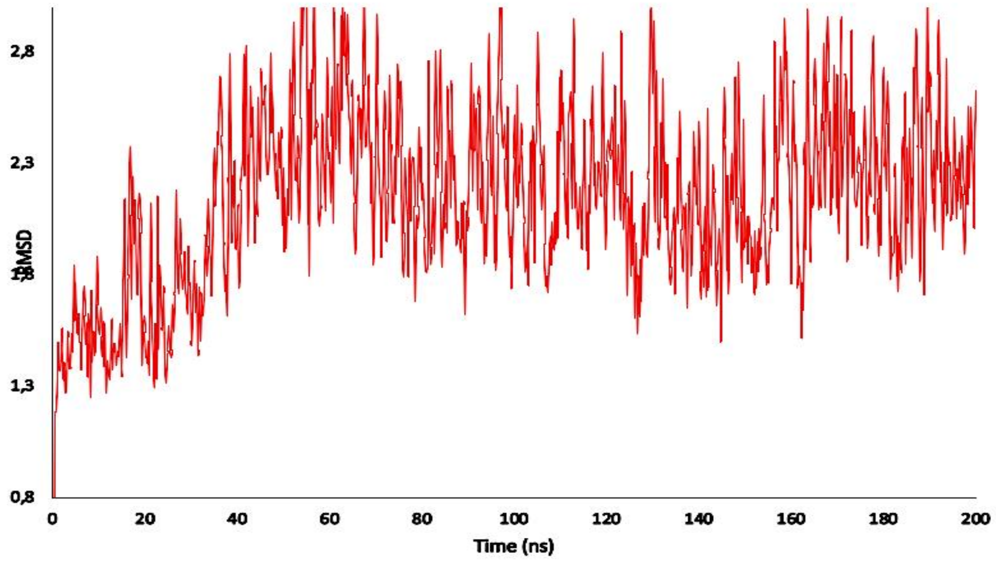

Figure S12. Visualization of root-mean-square deviation (RMSD) of the 4XO9 protein with the ligand native.

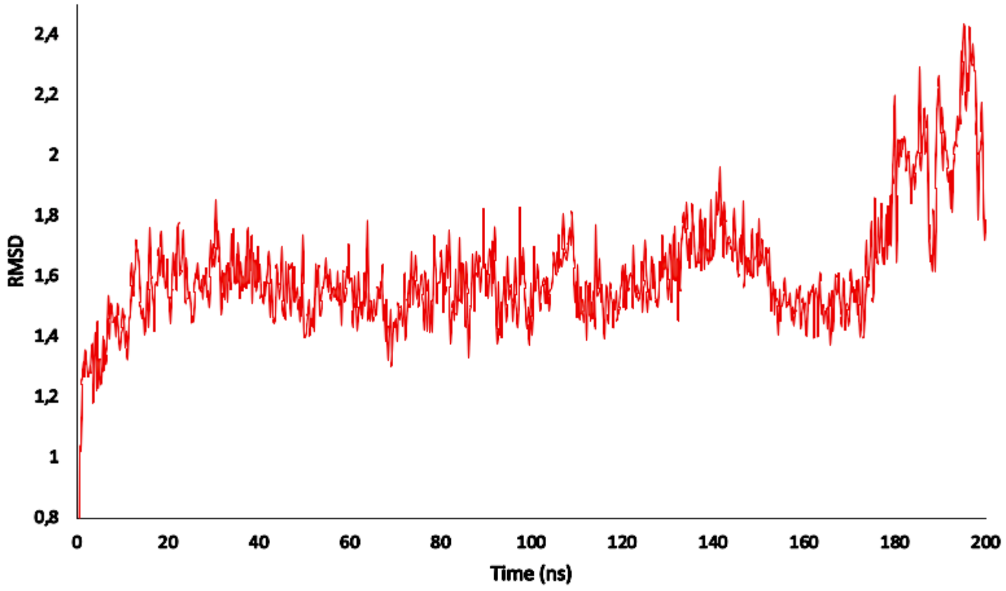

Figure S13. Visualization of root-mean-square deviation (RMSD) of the 4XO9 protein with the ligand b.

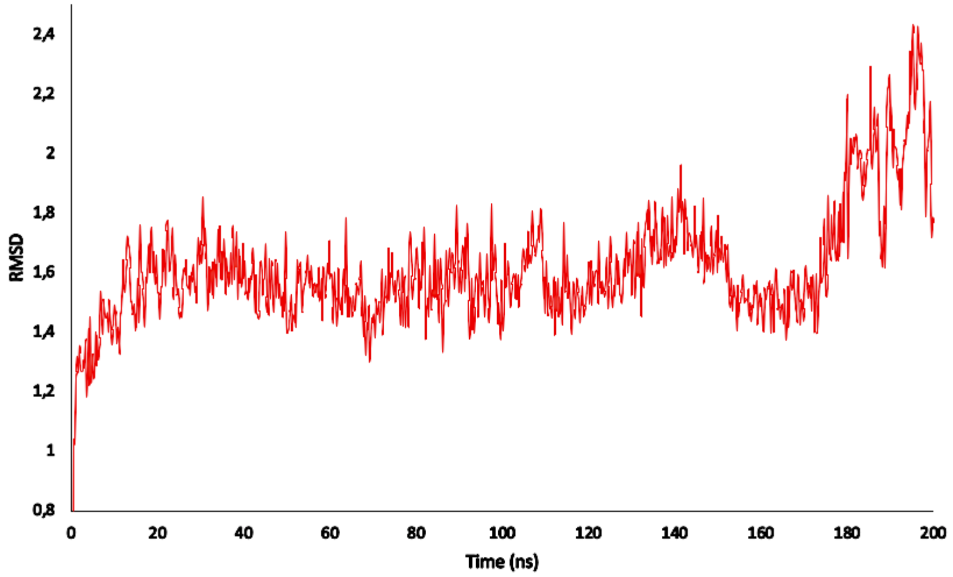

Figure S14. Visualization of root-mean-square deviation (RMSD) of the 4XO9 protein with the ligand c.

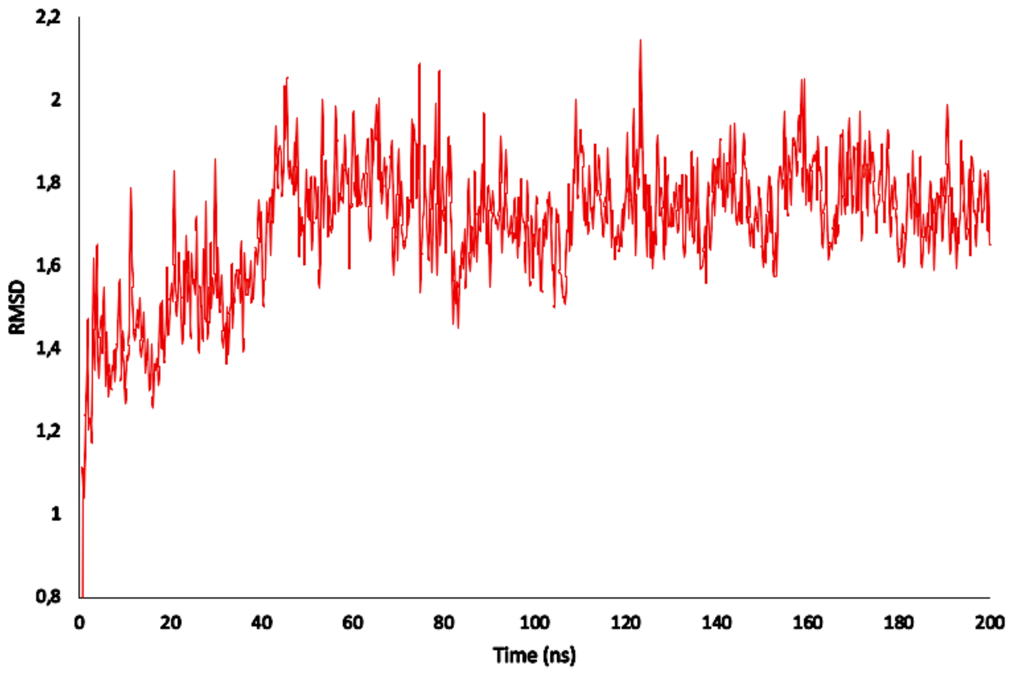

Figure S15. Visualization of root-mean-square deviation (RMSD) of the 4XO9 protein with the ligand d.

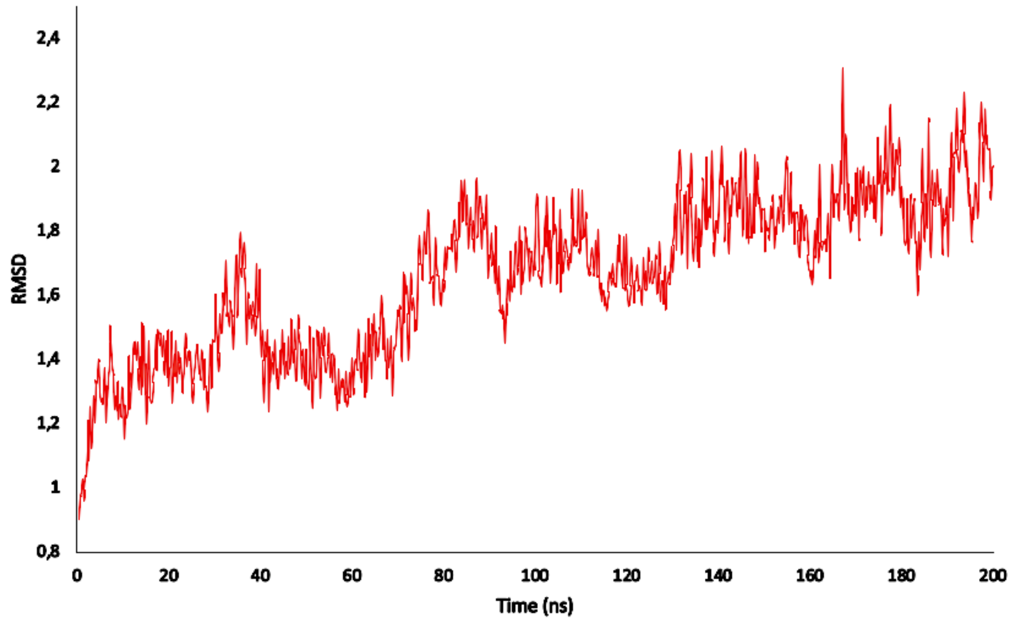

Figure S16. Visualization of root-mean-square deviation (RMSD) of the 4XO9 protein with the ligand e.

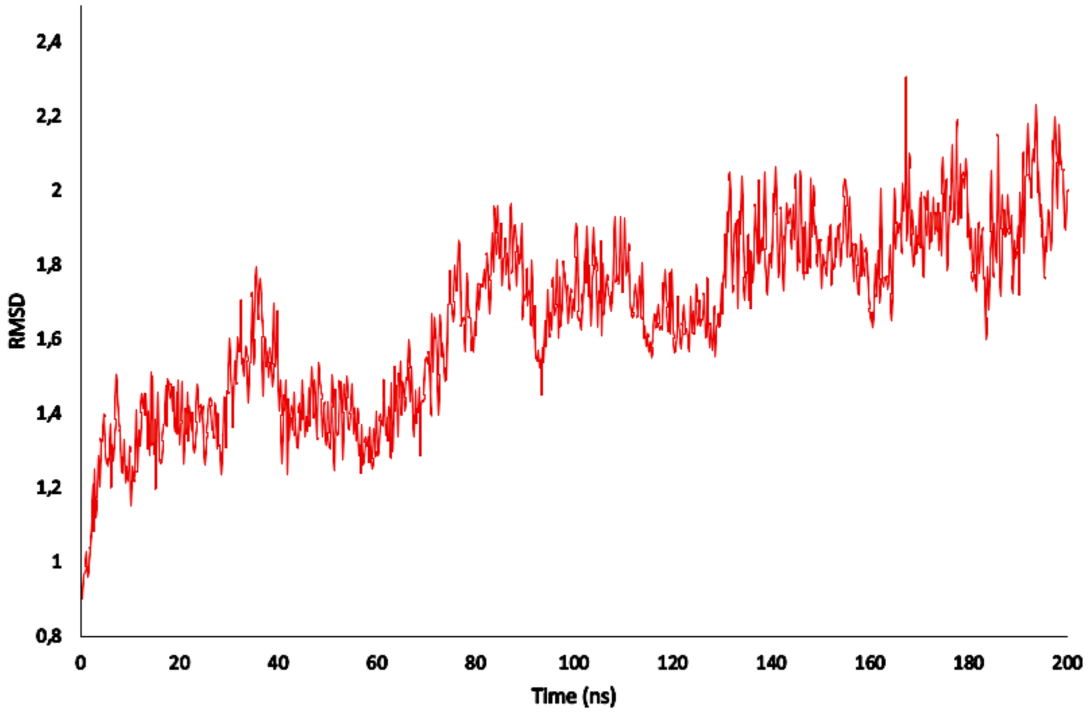

Figure S17. Visualization of root-mean-square deviation (RMSD) of the 4XO9 protein with the ligand f.

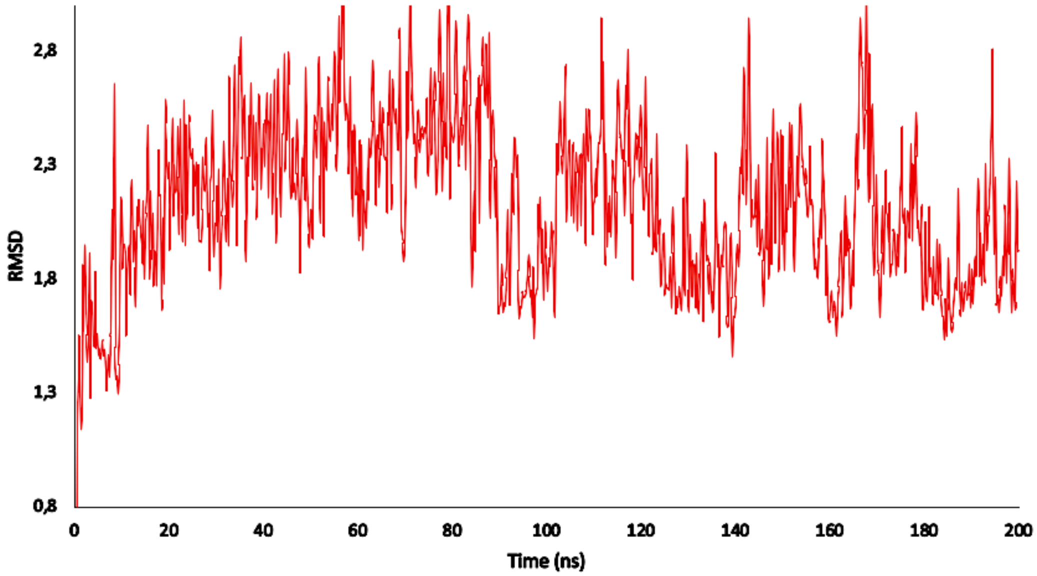

Figure S18. Visualization of root-mean-square deviation (RMSD) of the 4XO9 protein with the ligand g.

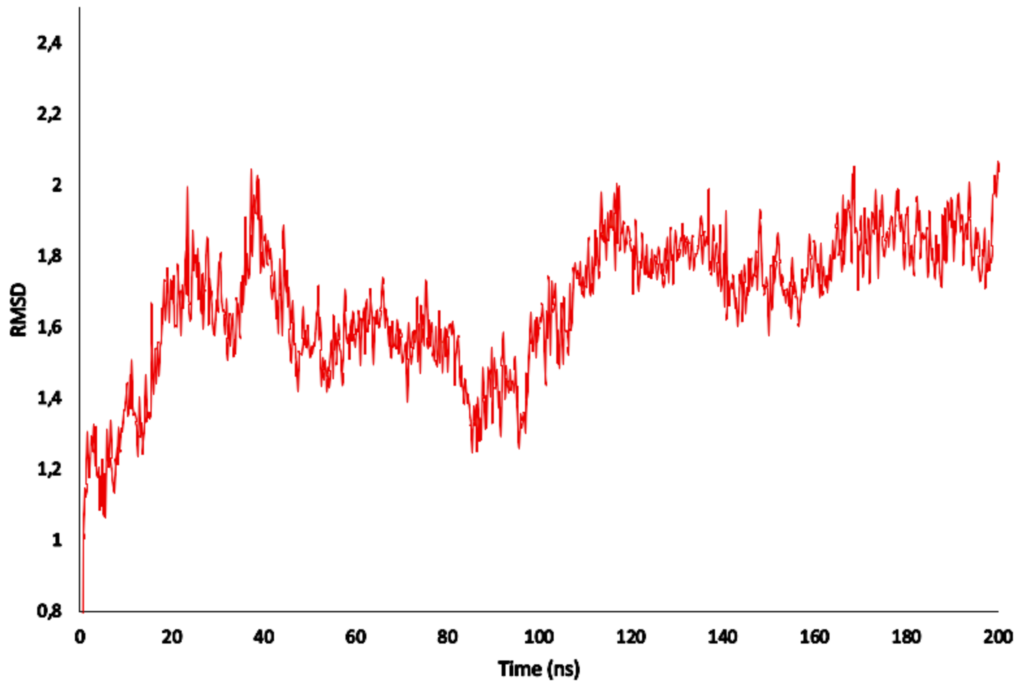

Figure S19. Visualization of root-mean-square deviation (RMSD) of the 4XO9 protein with the ligand h.

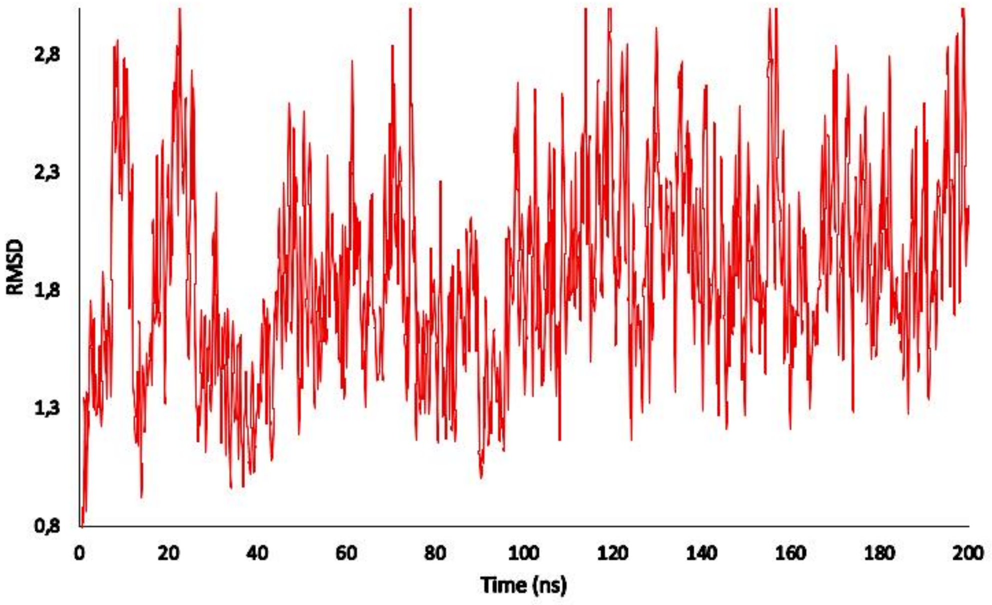

Figure S20. Visualization of root-mean-square deviation (RMSD) of the 4XO9 protein with the ligand i.

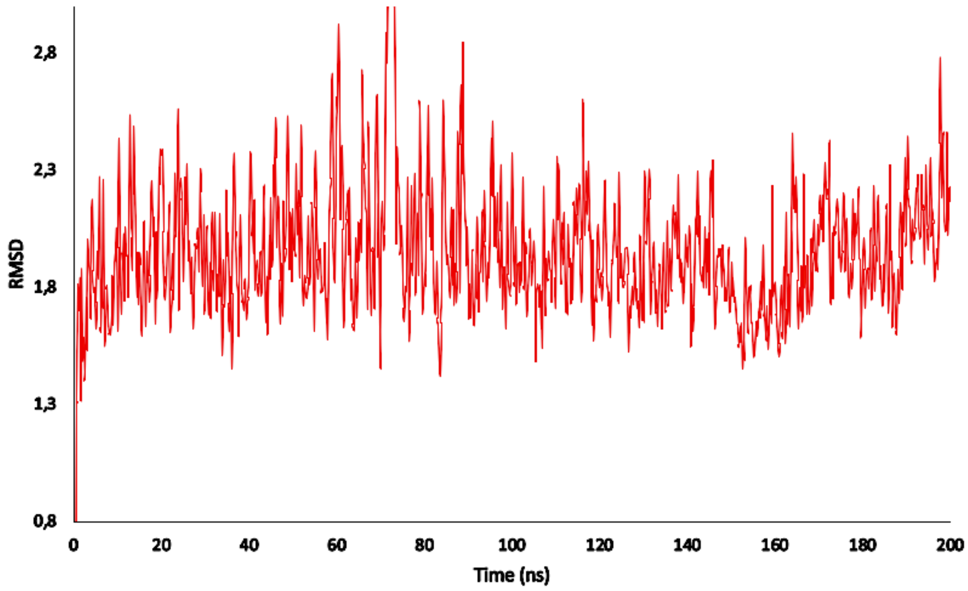

Figure S21. Visualization of root-mean-square deviation (RMSD) of the 4XO9 protein with the ligand j.

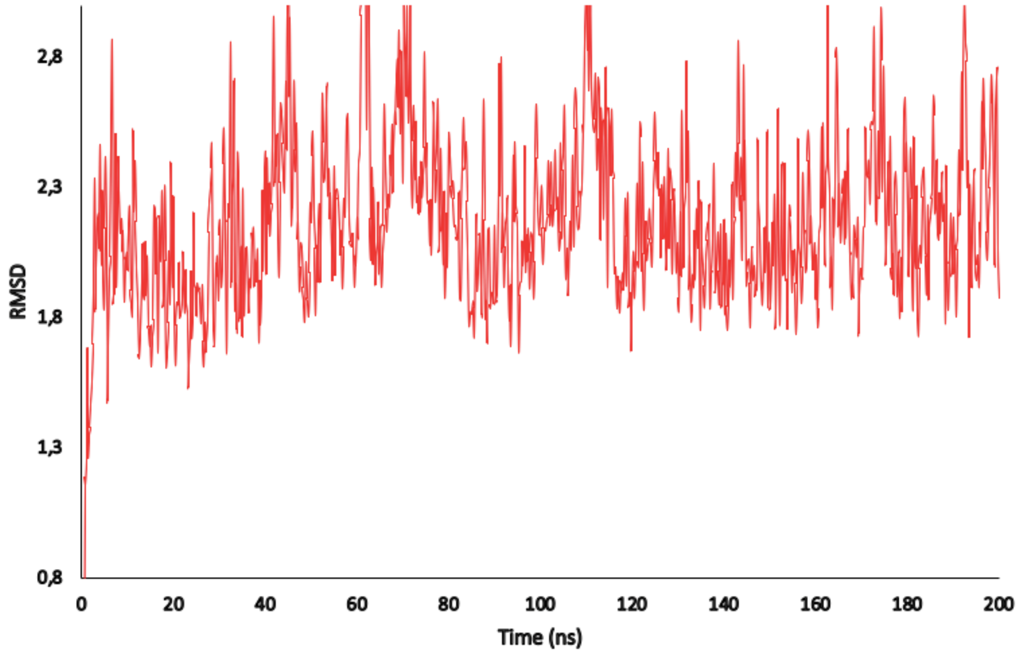

Figure S22. Visualization of root-mean-square deviation (RMSD) of the 4XO9 protein with the ligand k.
